# The Post-Acute Phase of SARS-CoV-2 Infection in Two Macaque Species Is Associated with Signs of Ongoing Virus Replication and Pathology in Pulmonary and Extrapulmonary Tissues

**DOI:** 10.3390/v13081673

**Published:** 2021-08-23

**Authors:** Kinga P. Böszörményi, Marieke A. Stammes, Zahra C. Fagrouch, Gwendoline Kiemenyi-Kayere, Henk Niphuis, Daniella Mortier, Nikki van Driel, Ivonne Nieuwenhuis, Richard A. W. Vervenne, Tom Haaksma, Boudewijn Ouwerling, Deborah Adema, Roja Fidel Acar, Ella Zuiderwijk-Sick, Lisette Meijer, Petra Mooij, Ed J. Remarque, Herman Oostermeijer, Gerrit Koopman, Alexis C. R. Hoste, Patricia Sastre, Bart L. Haagmans, Ronald E. Bontrop, Jan A. M. Langermans, Willy M. Bogers, Ivanela Kondova, Ernst J. Verschoor, Babs E. Verstrepen

**Affiliations:** 1 Biomedical Primate Research Centre (BPRC), Department of Virology, 2288 GJ Rijswijk, The Netherlands; Boszormenyi@bprc.nl (K.P.B.); fagrouch@bprc.nl (Z.C.F.); kayere@bprc.nl (G.K.-K.); niphuis@bprc.nl (H.N.); mortier@bprc.nl (D.M.); acar@bprc.nl (R.F.A.); mooij@bprc.nl (P.M.); remarque@bprc.nl (E.J.R.); oostermeijer@bprc.nl (H.O.); koopman@bprc.nl (G.K.); bogers@bprc.nl (W.M.B.); verstrepen@bprc.nl (B.E.V.); 2 Department of Parasitology, BPRC, 2288 GJ Rijswijk, The Netherlands; stammes@bprc.nl (M.A.S.); nieuwenhuis@bprc.nl (I.N.); vervenne@bprc.nl (R.A.W.V.); meijer@bprc.nl (L.M.); 3 Animal Science Department, BPRC, 2288 GJ Rijswijk, The Netherlands; vandriel@bprc.nl (N.v.D.); haaksma@bprc.nl (T.H.); ouwerling@bprc.nl (B.O.); adema@bprc.nl (D.A.); langermans@bprc.nl (J.A.M.L.); kondova@bprc.nl (I.K.); 4 Alternatives Unit, BPRC, 2288 GJ Rijswijk, The Netherlands; sick@bprc.nl; 5 Eurofins-Inmunología y Genética Aplicada (Eurofins-INGENASA), 28037 Madrid, Spain; alexis.hoste@uliege.be (A.C.R.H.); psastre@ingenasa.com (P.S.); 6 Department of Viroscience, Erasmus University Medical Center, 3015 GD Rotterdam, The Netherlands; b.haagmans@erasmusmc.nl; 7 Department of Comparative Genetics and Refinement, BPRC, 2288 GJ Rijswijk, The Netherlands; bontrop@bprc.nl; 8 Department of Biology, Theoretical Biology and Bioinformatics, Utrecht University, 3584 CH Utrecht, The Netherlands; 9 Unit Animals in Science and Society, Department of Population Health Sciences, Veterinary Faculty, Utrecht University, 3584 CL Utrecht, The Netherlands

**Keywords:** SARS-CoV-2, COVID-19, non-human primates, animal models, NHPs

## Abstract

The post-acute phase of SARS-CoV-2 infection was investigated in rhesus (*Macaca mulatta*) and cynomolgus macaques (*Macaca fascicularis*). During the acute phase of infection, SARS-CoV-2 was shed via the nose and throat, and viral RNA was occasionally detected in feces. This phase coincided with a transient change in systemic immune activation. Even after the alleged resolution of the infection, computed tomography (CT) and positron emission tomography (PET)-CT revealed pulmonary lesions and activated tracheobronchial lymph nodes in all animals. Post-mortem histological examination of the lung tissue revealed mostly marginal or resolving minimal lesions that were indicative of SARS-CoV-2 infection. Evidence for SARS-CoV-2-induced histopathology was also found in extrapulmonary tissue samples, such as conjunctiva, cervical, and mesenteric lymph nodes. However, 5–6 weeks after SARS-CoV-2 exposure, upon necropsy, viral RNA was still detectable in a wide range of tissue samples in 50% of the macaques and included amongst others the heart, the respiratory tract and surrounding lymph nodes, salivary gland, and conjunctiva. Subgenomic messenger RNA was detected in the lungs and tracheobronchial lymph nodes, indicative of ongoing virus replication during the post-acute phase. These results could be relevant for understanding the long-term consequences of COVID-19 in humans.

## 1. Introduction

Biomedical and clinical researchers have tried to define the different phases of a severe acute respiratory syndrome coronavirus 2 (SARS-CoV-2) infection in humans from various perspectives [1,2,3,4]. The general consensus is that the initial phase of infection is viremia or the progressive state in which immune responses determine if the person remains healthy or not. This phase, which takes about a week, is followed by the pneumonia phase that generally lasts no longer than three weeks after the initial infection. Then, a convalescent phase is initiated during which the infection resolves, and patients recover from the disease. Nevertheless, evidence is accumulating for the existence of an enlengthened recovery process, even for non-hospitalized patients [5,6,7]. Moreover, in this phase, chronic symptoms and possible recurrence of disease can be detected, known as long-COVID or post-acute COVID [7,8]. However, it is still unknown how long this recovery phase lasts.

To halt and control the coronavirus disease 2019 (COVID-19) pandemic, enormous efforts have been initiated worldwide to develop vaccines and antiviral compounds. To achieve these challenging goals, as well as to acquire fundamental knowledge of understanding the mode of infection and its associated pathologies, animal studies play a crucial role.

Various animal species have already proven their value in SARS-CoV-2 research, such as mice, hamsters, and ferrets [9,10,11,12,13,14,15,16,17]. Notwithstanding the great importance of rodent and ferret models, non-human primates (NHPs) have shown to play an especially pivotal role in COVID-19 research. NHPs are susceptible to infection with SARS-CoV-2 and share many immunological and pathological characteristics with humans [9,18,19]. This makes NHPs particularly suitable for preclinical evaluation of vaccines and antiviral or immunomodulatory compounds against SARS-CoV-2. The two most widely used NHP species in biomedical research in general, and also in COVID-19 research, are rhesus macaques and cynomolgus macaques. Both species had already featured a prominent role in research on the related coronaviruses that caused the SARS and MERS epidemics [20,21], and are also considered relevant NHP models for preclinical COVID-19 studies [9].

Several research groups [22,23,24] have investigated the heterogeneity in SARS-CoV-2 infection and disease progression in different NHPs species, but mostly, relatively short follow-up studies were published, sacrificing animals at day 21 post-infection, or earlier [22,25,26,27]. So far, little is known about the post-acute phase of SARS-CoV-2. In patients who died of COVID-19, viral RNA was found widely disseminated throughout tissues and organs, and a wide variety of pathologies was found in postmortem tissue. The majority of human infections however are asymptomatic or cause mild disease. This is also the case in NHP. Little is known about the long-term consequences of asymptomatic or mild infection in humans. Here, we took a unique approach and focused our research on the first weeks of the post-acute phase, after a mild to moderate SARS-CoV-2 disease course in two macaque species. We followed SARS-CoV-2 replication in rhesus and cynomolgus macaques and monitored the animals for signs of COVID-19-like disease symptoms. The macaques were infected in parallel with the same virus stock and the same dose and underwent a fully identical treatment. The course of infection was followed for up to six weeks using the same analyses, including the monitoring of virus-induced metabolic and anatomic findings with CT and PET-CT, and continuous telemetric recording of body temperature and physical activity of the animals. The animals were sacrificed to investigate histopathological tissue changes related to SARS-CoV-2, as well as to examine viral dissemination in organs.

## 2. Materials and Methods

### 2.1. Ethics and Biosafety Statement

All housing and animal care procedures took place at the Biomedical Primate Research Centre (BPRC) in Rijswijk. The BPRC is accredited by the American Association for Accreditation of Laboratory Animal Care (AAALAC) International and is compliant with the European directive 2010/63/EU as well as the “Standard for Humane Care and Use of Laboratory Animals by Foreign Institutions” provided by the Department of Health and Human Services of the US National Institutes of Health (NIH, identification number A5539-01). Upon positive advice by the independent ethics committee (DEC-BPRC), the competent national authorities (CCD, Central Committee for Animal Experiments) issued a project license (license AVD5020020209404). Approval to start was obtained after further assessment of the detailed study protocol by the institutional animal welfare body (AWB) (in Dutch: Instantie voor Dierenwelzijn, IvD). All animal handlings were performed within the Department of Animal Science (ASD) according to Dutch law. ASD is regularly inspected by the responsible national authority (Nederlandse Voedsel- en Warenautoriteit, NVWA), and the AWB.

### 2.2. Animals

Four Indian-origin rhesus macaques and four South-Asian-origin cynomolgus macaques were used in this study (Supplementary Table S1). All macaques were mature, outbred animals, purpose-bred, and housed at the BPRC. All selected animals were healthy males with bodyweight in the normal range. The rhesus macaques (R-codes) were 5 and 6 years old. Three of the four cynomolgus macaques (J-codes) were 4 years of age. In addition, one older cynomolgus macaque was added (Ji408005). The animals are described in detail in Supplementary Table S1. The animals were in good physical health with normal baseline biochemical and hematological values. All were pair-housed with a socially compatible cage-mate in cages of at least 4 m^3^ with bedding to allow foraging and were kept on a 12-h light/dark cycle. The monkeys were offered a daily diet consisting of monkey food pellets (Ssniff, Soest, Germany) supplemented with vegetables and fruit. Enrichment was provided daily in the form of pieces of wood, mirrors, food puzzles, and a variety of other homemade or commercially available enrichment products. Drinking water was available ad libitum via an automatic watering system. Animal care staff provided daily visual health checks before infection, and twice daily after infection. The animals were monitored for appetite, general behavior, and stool consistency. All possible precautions were taken to ensure their welfare and to avoid any discomfort to the animals. All experimental interventions (intratracheal and intranasal infection, swab collections, blood samplings, and (PET-)CTs) were performed under anesthesia.

### 2.3. Virus

The animals were infected with SARS-CoV-2 strain BetaCoV/BavPat1/BPRC_Berlin-1_P5. This strain was isolated from a patient who traveled from China to Germany, and an aliquot of a Vero E6 cell culture was made available through the European Virus Archive-Global (EVAg). The viral stock for the study was propagated on Vero E6 cells. For the infection, a 5th passage virus stock with a titer of 3.2 × 10^6^ TCID_50_/mL was prepared. TCID_50_ analysis was performed on Vero E6 cells in a 96-well format in triplicates. The virus stock was diluted 1:10 in culture medium as a starting dilution and then serially diluted. Plates were fixed on day 3 with 10% formalin and stained with crystal violet. The integrity of the virus stock used for infection was confirmed by next-generation sequencing analysis and the sequence was submitted to GenBank (MZ558051).

### 2.4. Experimental Infections and Post-Exposure Study Follow-Up

Three weeks before the experimental infection, a Physiotel Digital device (DSI Implantable Telemetry, Data Sciences International, Harvard Bioscience, UK) was implanted in the abdominal cavity of each animal. This device allowed the continuous real-time measurement of the body temperature and the animals’ activity remotely using telemetry throughout the study.

On day 0, all animals were exposed to a dose of 1 × 10^5^ TCID_50_ of SARS-CoV-2, diluted in 5 mL phosphate-buffered saline (PBS) (Supplementary Table S5). The virus was inoculated via a combination of the intratracheal route, just below the vocal cords, (4.5 mL) and intranasal route (0.25 mL in each nostril). Virus infection was monitored for 35 to 42 days, and during that period the animals were checked twice daily by the animal caretakers and scored for clinical symptoms according to a previously published though adapted scoring system [28] (Supplementary Table S2). A numeric score of 35 or more per observation time point was predetermined to serve as an endpoint and justification for euthanasia. Every time an animal was sedated, the bodyweight was measured. Blood was collected using standard aseptic methods from the femoral vein at regular time points post-infection (pi). In parallel, tracheal, nasal, and anal swabs were collected using Copan FLOQSwabs (MLS, Menen, Belgium). Swabs were placed in 1 mL MEM, supplemented with 0.5% bovine serum albumin (BSA), fungizone (2.5 μg/mL), penicillin (100 U/mL), and streptomycin (100 μg/mL) and directly transported to the BSL3 lab.

### 2.5. Biochemistry and Hematology

Clinical biochemistry was performed using a Vetscan VS2 Chemical analyzer (Zoetis Benelux, Capelle aan de IJssel, The Netherlands) with the use of the Comprehensive Diagnostic profile. This profile allows testing for alanine aminotransferase, albumin, alkaline phosphatase, amylase, calcium, creatinine, globulin, glucose, phosphorus, potassium, sodium, total bilirubin, total protein, and blood urea nitrogen. Hematology was done using a Vetscan HM5 Hematology analyzer (Zoetis Benelux, Capelle aan de IJssel, The Netherlands). C-reactive protein and D-dimer levels were measured using Cobas Integra 400 plus analyzer (Roche Diagnostics Nederland B.V., Almere, The Netherlands).

### 2.6. Detection of Viral RNA in Swabs, Blood, and Tissue

To determine the presence of SARS-CoV-2 RNA in post-mortem tissues, tissue samples were weighed and placed in gentleMACS M tubes (30 mg in 100 μL PBS) and dissociated using a gentleMACS Tissue Dissociator (protein01 program) (Miltenyi Biotec B.V., Leiden, The Netherlands). Next, the homogenized tissue was centrifuged for 10 min at 820× *g*, and 100 μL supernatant was used for RNA isolation. Viral RNA was isolated from plasma, swab sample supernatants, and cleared tissue homogenates using a QIAamp Viral RNA Mini kit (Qiagen Benelux BV, Venlo, The Netherlands) following the manufacturer’s instructions. Viral RNA was reverse-transcribed to cDNA using a Transcriptor First Strand cDNA Synthesis kit (Roche Diagnostics BV, Almere, The Netherlands). Viral genomic RNA was quantified by real-time quantitative RT-PCR specific for the RdRp gene of SARS-CoV-2, as described by Corman et al. [29]. Viral subgenomic messenger RNA (sgmRNA) was detected and quantified as previously described by Wölfel et al. [30]. Detection of sgmRNA was used as a as a proxy for the detection of replicating virus. For both assays, RNA standard curves were generated by in vitro transcription of the target regions. The number of RNA copies was calculated using the formula: # RNA copies = amount ∗ 6.022 × 10^23/^RNA length in nucleotides * 340. Both genomic and sgmPCR have a lower limit of quantification of 20 viral RNA copies per reaction. All PCR samples were analyzed in duplicate.

### 2.7. Imaging

Positron Emission Tomography (PET)-computed tomography (CT) and CT data were acquired on multiple time points post-infection using a MultiScan Large Field of View Extreme Resolution Research Imager (LFER) 150 PET-CT (Mediso Medical Imaging Systems Ltd., Budapest, Hungary) as described before [31]. Animals fasted overnight and were sedated with ketamine (10 mg/kg ketamine hydrochloride (Alfasan Nederland BV, Woerden, The Netherlands)) combined with medetomidine hydrochloride (0.05 mg/kg (Sedastart; AST Farma B.V., Oudewater, The Netherlands)) to induce sedation and muscle relaxation, both applied intramuscularly (IM). The animals were positioned head-first supine (HFS) with the arms up. After the scan, upon return to their home cage, atipamezole hydrochloride (Sedastop, AST Farma B.V., Oudewater, The Netherlands, 5 mg/mL, 0.25 mg/kg) was administrated IM to antagonize medetomidine.

#### 2.7.1. PET-CT

The PET-CT images were acquired under mechanical ventilation in combination with a forced breathing pattern. For anesthetic maintenance, a minimum alveolar concentration of isoflurane (iso-MAC) of around 0.80–1.00% was used. The body temperature of the animal was maintained by using the Bair Hugger (3M™, St Paul, MN, USA) supplied with 43 °C airflow. Typically, around 100 MBq of ^18^F-FDG was applied intravenously (GE Healthcare, Leiderdorp, The Netherlands). A 15-min static PET was acquired of the lungs 45 min after injection. Afterward, the emission data was iteratively reconstructed (OSEM3D, 8 iterations, and 9 subsets) into a single frame PET image normalized and corrected for attenuation, scatter, and random coincidences using the reference CT and corrected for radioactive decay. The analysis was performed in VivoQuant 4.5 (Invicro, Boston, MA, USA). Lung lesions were discriminated based on lung density as defined by Hounsfield Units (HU) on CT. Lymph node uptake was quantified with the use of a lower threshold standard uptake value (SUV) of 2.0 to discriminate activated lymph nodes from the surrounding tissue. Two anatomical and two molecular output parameters were generated; volume, anatomical density in HU, average standardized uptake value (SUVmean), and a maximum SUV corrected for scatter and random coincidences (SUVpeak). In this way, a full overview of the results could be obtained with minimal inter and intra-observer variation [31].

#### 2.7.2. Gated-CT

In order to mitigate motion artifacts and to allow a non-invasive CT, retrospective gating was applied during CT. The respiratory amplitude was detected with a gating pad placed next to the umbilicus. For the final reconstruction, the inspiration phases were exclusively used and manually selected. A semi-quantitative scoring system for chest CT evaluation was used to estimate SARS-CoV-2-induced lung disease [32,33,34]. Quantification of the CTs was performed independently, by two experienced imaging scientists based on the lobes; the middle and accessory lobe of the right lung were combined. The degree of involvement in each zone was scored as: 0 for no involvement, 1 for <5%, 2 for 5–24%, 3 for 25–49%, 4 for 50–74%, and 5 for >74% involvement. An additional increase or decrease of 0.5 was used to indicate alterations in CT density of the lesions. By using this scoring system, a maximum score of 30 could be reached for the combined lobes per time point.

### 2.8. Cytokine and Chemokine Analysis

Cytokine and chemokine concentrations in sera of infected macaques, including IL-1β, IL-6, CCL11 (Eotaxin), CXCL10 (IP-10), CXCL11 (I-TAC), CCL2 (MCP-1), CXCL9 (MIG), CCL3 (MIP-1α), CCL4 (MIP-1β), CCL5 (RANTES), CXCL8 (IL-8), TNF-α, and IFN-γ, were determined using the LEGENDplex™ NHP Chemokine/Cytokine Panel (13-plex) (Biolegend, San Diego, CA, USA) essentially according to manufacturer’s instruction. Samples were measured on an LSRII FACS machine (BD Biosciences, Vianen, The Netherlands) and analyzed by using company software.

### 2.9. Serology

Antibodies to SARS-CoV-2 in sera were analyzed using different enzyme-linked immunosorbent assays. A double recognition enzyme-linked immunosorbent assay (DR-ELISA) that detects total immunoglobulins targeted to the SARS-CoV-2 nucleoprotein (N) protein was used, as described by Hoste et al. [35] (INgezim COVID19 DR; Eurofins-INGENASA, Madrid, Spain). In addition, an ELISA that detects immunoglobulins elicited to the full-length spike (S) protein, and an assay that specifically detects antibodies directed to the receptor-binding domain (RBD) of the S protein were applied in this study.

For the analysis of anti-S protein antibody responses, Greiner half-area ELISA plates were coated overnight with 1 µg/mL of the monomeric full-length Spike or Spike RBD proteins (both antigens expressed in insect cells; AdapVac, Copenhagen, Denmark) in PBS. Plates were washed and blocked for one hour with PBS Tween-20 (0.05%) + BSA (3% m/v). Serial dilutions of sera were made in PBS Tween-20 (0.05%) + BSA (1% m/v) (PBS-TB) and added to the plate in duplicate. As a standard reference, a monoclonal antibody specific for the SARS-CoV-2 RBD was used, diluted in PBS-TB to an initial IgG concentration of 500 ng/mL, and diluted in the ELISA plate in a two-fold series over seven wells (Range 500 to 0.007813 ng/mL). Standards were run in duplicate. The reference standard and sera were incubated for one hour at 37 °C, and subsequently washed with PBS Tween-20 (0.05%). Goat-anti-human (H+L) IgG-HRP (Thermo Fisher Scientific, Waltham, MA, USA) was added to the plate and incubated for one hour at 37 °C after which the plates are washed and TMB substrate was added. The reaction was stopped after 15 min by the addition of 0.2 M H_2_SO_4_. Plates were then read at 450 nm on a SpectraMax M5 plate reader (Molecular Devices, LLC., San Jose, CA, USA), and OD values were saved as Excel files. A four-parameter curve was fit to the standard, and IgG concentrations in the samples were calculated from the OD values using the parameters as estimated from the four-parameter fit. Only OD values in the linear part (i.e., parallel to the standard curve) of the dilution curve were used for the samples. Within sample coefficient of variation (CV) for the included OD values usually was below 25%.

To detect IgM, the same protocol was followed, but the secondary antibody used was an anti-human IgM Peroxidase-labelled antibody produced in goats (SouthernBiotech, Birmingham, AL, USA).

### 2.10. Necropsy and Histological Analysis

After euthanasia, necropsies were performed according to a standard protocol. The samples were fixed by immersion in 10% neutral-buffered formalin, routinely processed into paraffin blocks, cut into 4 µm tissue sections, stained with Hematoxylin and Eosin (HE), and examined microscopically. Subsequent tissue sections on all seven pulmonary lobes of each animal were used for special stains such as Mason’s Trichrome staining for the presence of collagen and Gram staining for the detection of bacteria. In parallel, samples from the same organs were collected for virus detection by real-time PCR and for immune-histochemical (IHC) analyses.

### 2.11. Immunohistochemistry

The SARS-CoV-2 antigen was detected with an anti-SARS-CoV-2-nucleoprotein monoclonal antibody (clone E16C; ThermoFisher, MA, USA), and type II pneumocytes were identified with the anti-Thyroid Transcription Factor-1 (TTF-1) antibody (clone 8G7G3/1, Dako, Denmark). Both were used in combination with a Roche Optiview DAB IHC kit in a Ventana Benchmark Ultra immunostainer (Roche, Basel, Switzerland). Hematoxylin and Eosin (HE) staining was used for general morphology. Slides were assessed in a blind manner.

## 3. Results

### 3.1. Infection of Macaques with SARS-CoV-2

**Figure 1:**
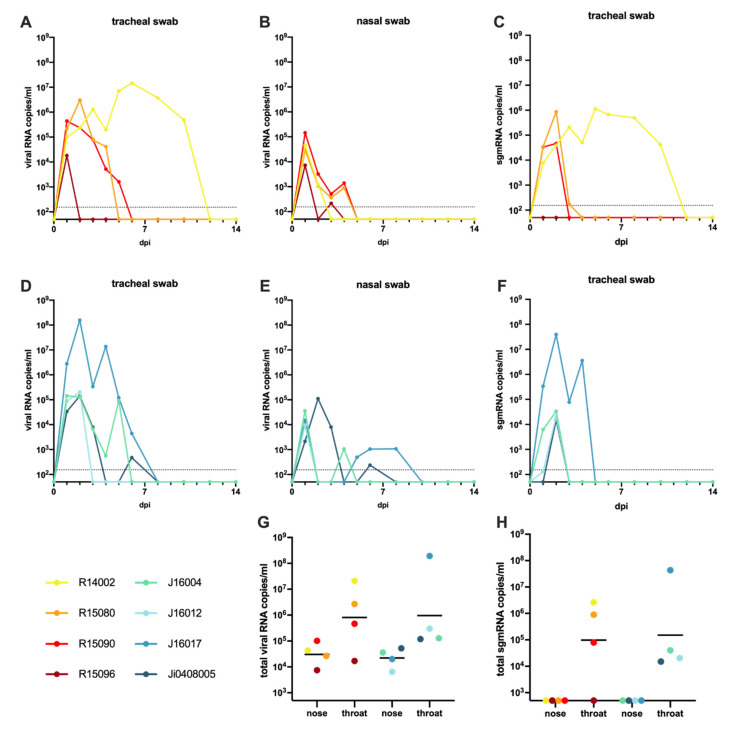
Virus loads in swab samples of macaques following SARS-CoV-2 infection. Viral RNA and subgenomic messenger RNA quantification in tracheal and nasal swabs of rhesus and cynomolgus macaques by qRT-PCR. The limit of quantification is indicated by the dotted horizontal line. Swabs after 14 dpi were all negative in PCR analysis and are therefore not shown for improved clarity. Viral RNA levels in tracheal (**A**) and nasal swabs (**B**), and sgmRNA levels in tracheal swabs (**C**) of rhesus macaques. Viral RNA levels in tracheal (**D**) and nasal swabs (**E**), and sgmRNA levels in trachea swabs (**F**) of cynomolgus macaques. The limit of quantification is indicated by the dotted horizontal line. Total virus loads (**G**) and sgmRNA loads (**H**) in throat and nose samples of macaques throughout the study. Horizontal bars represent geometric means. The sum of the viral copies was calculated rather than the area under the curve (AUC), as AUC interpolates for time points when virus loads were not determined. The color coding used for each individual animal as shown in the figure is used throughout the manuscript to denote the same individual. Rhesus macaques are indicated by yellow to red colors; cynomolgus macaques by green to blue colors.

Following the administration of the virus in the upper trachea and nose, viral RNA was detectable in the tracheal and nasal swabs of all monkeys at day 1 post-infection (pi). The time frame in which viral RNA was detected varied considerably from one day (tracheal swab macaque R15096) to up to ten days in macaque R14002. (Figure 1A,B,D,E, Supplementary Table S3A). The individual variation of SARS-CoV-2 RNA levels detected in the macaques was, regardless of species, substantial. Peak viral RNA levels in the throat varied between 1.7 × 10^4^ genome equivalent (GE)/mL (R15096; day 1 pi) and 1.8 × 10^8^ GE/mL (J16017; day 2 pi). Peak viral loads detected in nasal swabs were generally lower than in the throat samples and did not exceed 9.5 × 10^4^ GE/mL (R15090; day 1 pi). The high RNA loads measured in the first two days post-infection are suggestive of residual RNA from the original inoculum still being present. However, nasal swabs from five out of eight animals tested positive again after one or more days of undetectable levels. Cynomolgus macaque J16017 was positive in the nose at day 1 pi, then had no detectable viral RNA for a period of three days, but later the animal became again positive in the nose swabs for three consecutive days. Furthermore, R15096, J16004, J16012, and Ji408005 became PCR-positive again after one or more days without detectable viral RNA. The total viral RNA production detected in nose and throat samples was comparable over time for both species (Figure 1G).

The analysis of viral subgenomic messenger RNA (sgmRNA), which is considered to better reflect actual virus replication [36], confirmed infection in 7 out of 8 macaques. Only rhesus macaque R15096, which already displayed relatively low levels of viral RNA (Figure 1A,B) was negative for sgmRNA in both throat and nose swabs at all time points. In the remaining animals, sgmRNA levels were exclusively detected in the tracheal swabs, while no evidence of replication was found in the nasal swab samples (Figure 1C,F,H, Supplementary Table S3B).

In the anal swabs, viral RNA was rarely detected. Few macaques irregularly tested positive in the PCR test, with a low maximum viral RNA load of 3 × 10^3^ GE/mL at day 1 pi (J16017). No sgmRNA was detected in anal swabs or blood samples, indicating the absence of active virus replication in the blood and intestinal tract in the animals.

### 3.2. Body Temperature, Activity, Clinical Symptoms, and Blood Parameters after SARS-CoV-2 Infection

Body temperature and activity of each animal were continuously monitored using telemetry during the entire study. Modestly elevated body temperatures were measured in both macaque species during the first two weeks after infection as compared to later time points. Whereas three out of four cynomolgus macaques peaked as early as day 2 pi, the elevation in body temperature in two out of four rhesus macaques occurred later on day 8 pi. The temperature curves for the individual animals, depicted in Supplementary Figure S1 show a return to baseline temperatures for all individuals after the initial phase of infection.

**Figure 2:**
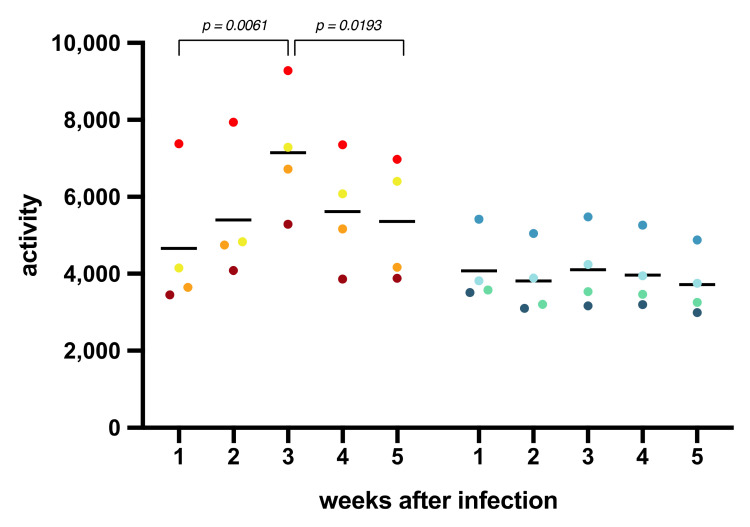
Cumulative activity scores. The activity of the animals was measured by telemetry throughout the study. The cumulative activity scores for rhesus and cynomolgus macaques were calculated as the area under the curve (AUC) per week. Measurements done during biotechnical handlings were omitted from the calculations.

The cumulative activity scores per week were calculated as the area under the curve (Figure 2). A significantly lower activity during the first week compared to the third week after infection was observed in the rhesus macaques (paired *t*-test; *p* = 0.0061) suggesting an impact of SARS-CoV-2 infection on the general well-being of the animals. Notably, in the last 2 weeks of the study, activity scores decreased again for all rhesus macaques (*p* = 0.0193). In contrast with these observations, the activity scores of the cynomolgus macaques remained unaffected throughout the study, which implies a species-related difference in reaction to SARS-CoV-2 infection. Activity curves for the individual animals are documented in Supplementary Figure S2.

A scoring list was used to enumerate overt clinical symptoms that may be caused by the SARS-CoV-2 infection (Supplementary Table S2). The cumulative clinical score per week of each animal was calculated (Supplementary Figure S3). In the first week after infection, the scoring of the four rhesus and two cynomolgus macaques did not exceed 25 (of maximum 770 per week), while for the remaining two animals a higher, but statistically not significant, median score was calculated. In the subsequent weeks after infection, none of the animals showed clinical signs suggestive of COVID-19.

Blood samples were analyzed for changes in cell subsets and in biochemical parameters upon SARS-CoV-2 infection (Supplementary Figure S4). Data were related to a set of normal (standard) values derived from a large group of uninfected, healthy macaques from the same breeding colony. C-reactive protein levels, which are increased in COVID-19 patients with pneumonia [37], were not elevated in the infected macaques. In humans, acute kidney injury has been related to SARS-CoV-2 infection [38,39], and elevated levels of serum creatinine and blood urea were detected in 10–15% of a cohort of COVID-19 patients [40]. Hence, we measured creatinine and urea levels in blood samples at multiple days post-infection but did not find evidence of kidney malfunction in the macaques. Equally, depending on the severity of the disease, blood coagulation disorders, such as highly elevated D-dimer levels, have been reported for patients [41,42], but no elevated D-dimer levels were measured in either macaque species. Elevated levels of glucose and alanine transferase were measured in the first week pi in the blood of most animals, and amylase was increased in one rhesus macaque, R15080. Also, for other blood cell subsets, no significant deviations from the normal values were seen in the infected monkeys.

### 3.3. Humoral Immune Response to SARS-CoV-2 Infection

**Figure 3:**
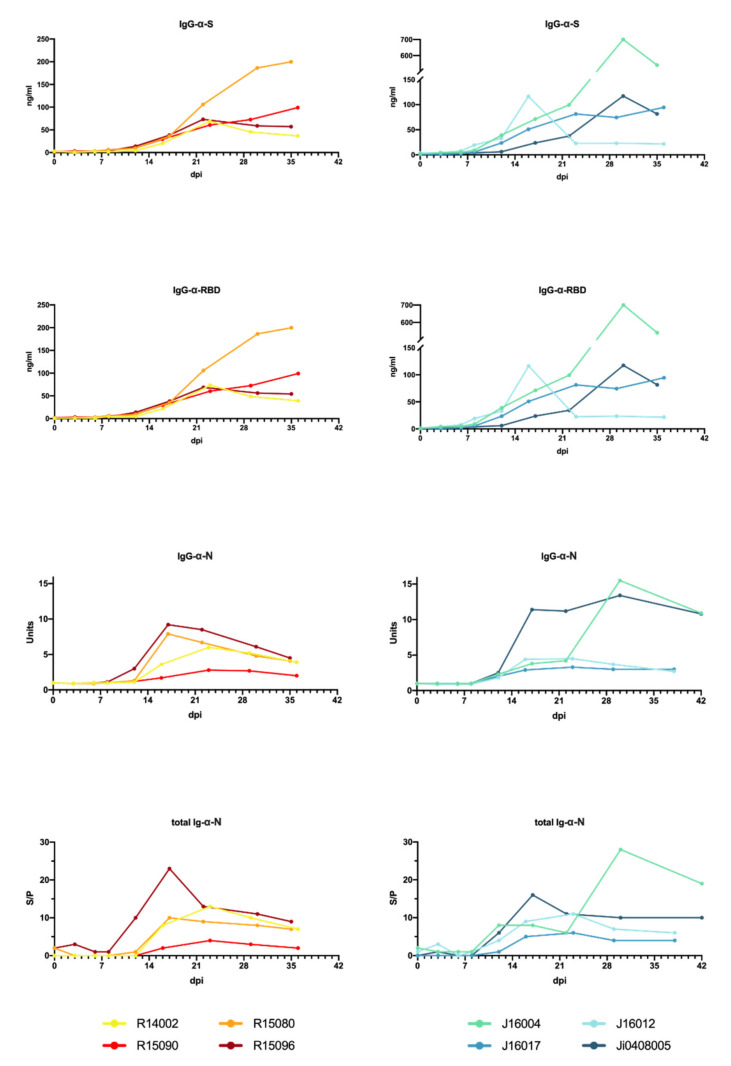
Development of a SARS-CoV-2 antibody response in rhesus and cynomolgus macaques. The humoral immune response was determined using an anti-S IgG ELISA, an anti-RBD ELISA, a serological test to detect IgG directed to the N protein, and a DR-ELISA measuring the total antibody response to N (left to right). Results of DR-ELISA are shown as S/P: sample to positive control ratio: $S/P=\frac{test\;sample-mean\;negative\;control}{mean\;positive\;control-mean\;negative\;control}$ and $Units=\frac{test\;sample\;at\;x\;dpi}{test\;sample\;at\;0\;dpi\;}$.

Humoral immune responses to the viral spike (S) and nucleoprotein (N) proteins were readily detectable after infection (Figure 3 and Supplementary Figure S5).

IgG titers to the ectodomain of the spike protein were first detectable 6–8 days pi. Titers continued to rise and reached a plateau after approximately 4 weeks. IgG levels in cynomolgus macaques showed more variation compared to rhesus macaques. Especially, the IgG kinetics in J16012 deviated from the other animals. In this animal, a peak in IgG was observed 17 days pi, and titers rapidly declined thereafter, and then remained consistently low throughout the rest of the study period. Overall, IgG was detectable slightly earlier in cynomolgus macaques compared to rhesus macaques, but the differences were not statistically relevant. In addition to the total anti-S IgG, IgG directed to the receptor-binding domain (RBD) were analyzed. The development of anti-RBD IgG fully mirrored the IgG antibodies binding to the entire S protein. IgM directed to the full-length S protein and the RBD were also measured (Supplementary Figure S5), and titers developed around the same time point post-infection as IgG titers. Overall, IgM titers in cynomolgus macaques were higher than in rhesus macaques, except for R15090. Anti-RBD IgM in rhesus macaques remained low in the study period, while in three out of four cynomolgus macaques rising anti-RBD IgM titers were measured.

In addition to the immunoglobulin responses to the spike protein, antibody responses to the nucleoprotein (N) were measured. Total immunoglobulin (Ig) in sera was determined by DR-ELISA. Ig responses became evident between day 10 and 12 pi. For both rhesus as well as cynomolgus macaques, a peak in levels was measured between 12 and 23 days pi, except in Ji0408005, where the peak was at day 30 pi.

Total Ig was reflected by IgG directed to the N protein. Notably, IgM responses to N were virtually undetectable in the longitudinal serum samples. Only in one animal, cynomolgus macaque J16012, IgM titers were transiently detectable (Supplementary Figure S5).

### 3.4. Cytokine and Chemokine Measurements in Sera of Infected Macaques

**Figure 4:**
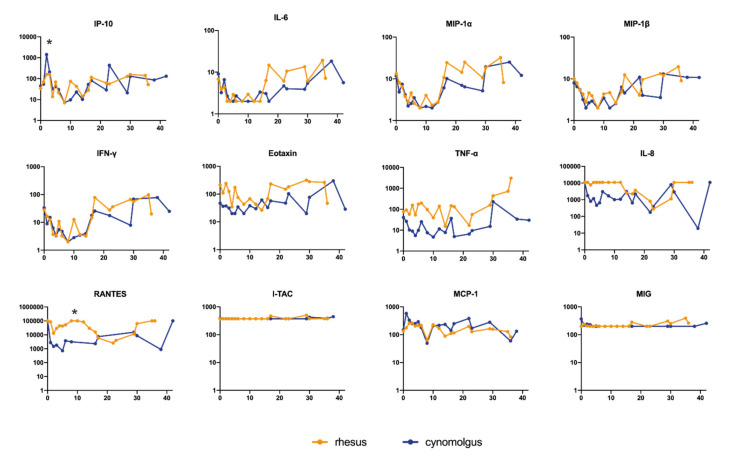
Cytokine and chemokine levels in peripheral blood after SARS-CoV-2 infection. Longitudinal sera collected after SARS-CoV-2 infection were tested. Results were expressed as pg/mL. Statistically significant differences in the geometric mean between rhesus and cynomolgus macaques are indicated with *.

We measured a panel of 13 cytokines and chemokines to characterize the inflammatory response triggered by SARS-CoV-2. In general, the cytokine and chemokine responses reflect two phases. The first phase is characterized by a transient decrease in IP-10, IL-6, MIP-1α, MIP-1β, and IFN-γ levels (Figure 4; Supplementary Figure S6 individual levels) while others were maintained at a stable level (Eotaxin, TNF-α, and IL-8). This trend was observed in both species, except for IP-10 and RANTES. At day 2 pi, the serum levels of IP-10 were nine-fold (95% CI 2.72–29.89) higher in the cynomolgus macaques than in rhesus macaques (*t*-test; *p* = 0.005). Also, the response pattern of RANTES was different between the two species. In cynomolgus macaques, RANTES rapidly decreased after exposure to SARS-CoV-2 and slowly returned to normal levels at the end of the follow-up period. In rhesus macaques, a transient dip in RANTES levels was observed early after infection. The levels returned to normal within 8 days pi but started to decline again in the following days, this is referred to as the second phase of the infection. At the end of the study, RANTES levels were back to normal in both species. MCP-1 (CCL2) showed a variable increase in two out of four rhesus macaques and in all cynomolgus macaques (Supplementary Figure S6).

### 3.5. Development of Lung Lesions and Lymph Node Activation during SARS-CoV2 Infection

**Figure 5:**
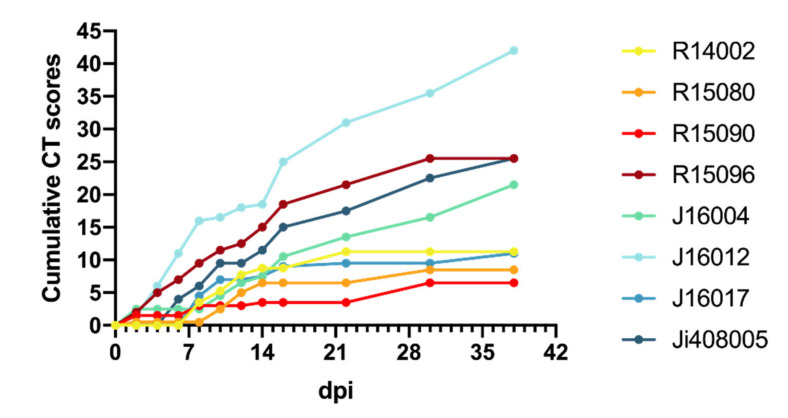
Cumulative CT scores. Cumulative CT scores for each animal were calculated based on the CT scores depicted in Table 1.

Chest CTs of the macaques after infection revealed several manifestations of COVID-19 with a variable time course and lung involvement (Table 1). The most common lesions found were ground-glass opacities (GGO), consolidations, and crazy paving patterns [3,33,43]. Lung lesions were already seen in the first CT obtained two days after infection (max. CT score 2.5/30), in five out of eight monkeys, three rhesus, and two cynomolgus macaques. Thereafter, lung involvement was seen in most animals and CT scores increased. Around days 8 and 10 pi, lung lesions had become manifested in all animals, and in several macaques, the scores had increased. Individual differences were considerable, varying from low scores on irregular time points (R15090) to high CT scores almost throughout the entire study period (J16012). The average cumulative CT score at day 38 pi was 12.9 for the rhesus macaques and 25 for the cynomolgus macaques (Mann–Whitney U test, *p* = 0.1702) with an average increase of 0.44 and 0.71 per day for rhesus and cynomolgus macaques, respectively (Mann–Whitney U test, *p* = 0.5036) (Figure 5).

For visualization and quantification of lymph node activation in the mediastinum, the tracheobronchial area, four (rhesus macaques) or five (cynomolgus macaques) weekly PET-CTs were obtained over a period of 5 weeks, starting at day 8 pi. The PET-CTs showed increased metabolic uptake in at least one tracheobronchial lymph node for all the macaques at all time points. The volume of the lymph nodes associated with this increased uptake ranged from 27 to 2350 mm^3^ (mean 620 mm^3^, std 534 mm^3^) (Supplementary Figure S7). On average, a lymph node is defined as enlarged when the volume is at least 194 mm^3^ [44]. The volume, average uptake (SUVmean), and the maximum uptake (SUVpeak) of the lymph nodes decreased simultaneously, while the density stayed approximately the same over time for most animals, except for one animal (J16012). This indicates that even in the enlarged lymph nodes no form of necrosis was initiated over time, as otherwise the density would have changed, the maximum uptake would be unaffected, and the average uptake would have decreased. No differences were observed between rhesus and cynomolgus macaques apart from the anatomical density represented by the Hounsfield units (HUs) (*p* < 0.001) (Supplementary Figure S7). Based on control scans in healthy animals (*n* = 24) we determined a negative correlation (r = 0.7154, *p* ≤ 0.0001) between weight and anatomical density of, at least, the subcarinal area in macaques, which can explain this difference.

**Figure 6:**
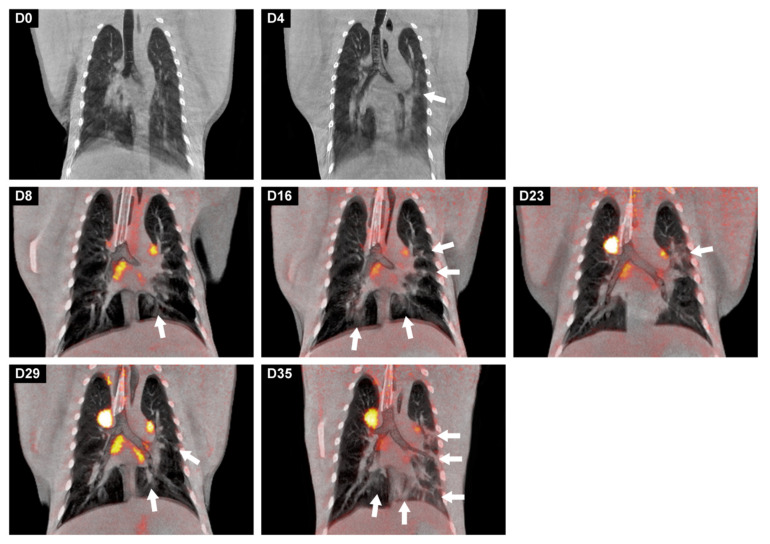
Longitudinal development of both lung lesions and metabolic activity in tracheobronchial lymph nodes of J16017 over time after a SARS-CoV-2 infection. Representative coronal slices with a thickness of 3 mm of J16017 were used for visualization. On days 0 and 4, only CT images were obtained, afterwards, until day 35, CT was combined with PET. The location of the lesions, marked with arrows, differed almost per time point, but are most prominently localized in the left lung on day 35.

From the longitudinal analysis, we conclude that the differentiation of lung lesions can be performed solely on the CT results, as the metabolic activity was only minimally increased on the PET images. An interesting finding is that the location of the lung lesions was not fixed but varied per time point, for instance, in macaque J16012. This animal showed the most prominent lesions in both lungs. The longitudinal PET-CT follow-up (Figure 6) showed two “waves” of the lymph nodes’ activation. The first peak on day 8 and another peak on day 29 of which the explanation is unknown. At the time point of euthanasia (day 35), activation of tracheobronchial lymph nodes was still observed which is indicative for SARS-CoV-2 induced pathogenesis in the post-acute or convalescent phase of the infection.

### 3.6. Gross Pathology

Rhesus macaques R15080 and R15096 were euthanized on day 35 pi, and animals R15090 and R14002, on day 36 pi. The cynomolgus macaques J16012 and J16017 were euthanized on day 38 pi and animals J16004 and Ji0408005 on day 42 pi.

Macroscopically, the lungs of the rhesus and cynomolgus macaques appeared mostly unremarkable. One rhesus macaque (R15096) had few small foci with hyperemia at the dorsal aspects of the caudal left and right lung lobes, and in three cynomolgus macaques, the lungs had similar foci of hyperemia, confined also to the caudal pulmonary lobes. The gross examination of extrapulmonary organs revealed mildly to moderately enlarged cervical and mesenteric lymph nodes. The rest of the organs were macroscopically unremarkable.

### 3.7. Wide-Spread Presence of Viral RNA in Post-Mortem Tissue Samples, and Evidence of Active Virus Replication in the Respiratory Tract of Infected Macaques

**Figure 7:**
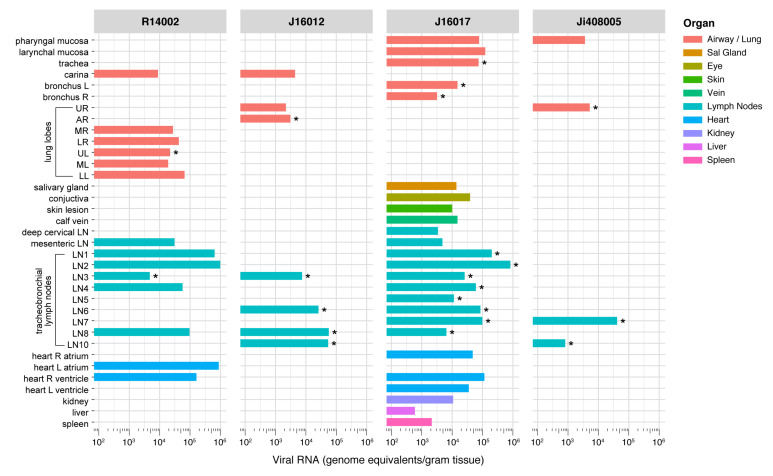
Viral RNA loads in tissues of SARS-CoV-2-infected rhesus macaques (R14002) and cynomolgus macaques (J16012, J16017, and Ji408005). Viral RNA was quantified by qRT-PCR and calculated per gram of tissue. Only animals with viral RNA-positive tissues are shown. On the *y*-axis, the tissues are listed, on the *x*-axis the RNA copies per g tissue. Tissues in which no RNA was observed in any of the animals are not shown. The tissues are grouped by color codes as indicated on the right. Tissues that tested positive for sgmRNA are indicated with *. Abbreviations: L, left; R, right; LN, lymph node; UR, upper right lung lobe; AR, accessory right lung lobe; MR, middle right lung lobe; LR, lower right lung lobe; UL, upper left lung lobe; ML, middle left lung lobe; LL, lower left lung lobe.

Post-mortem tissue samples were analyzed for the presence of viral RNA (Figure 7, Supplementary Table S3) and subgenomic messenger RNA (Table 2). After euthanasia, viral RNA was detected in tissue samples of four out of eight animals, one rhesus (R14002) and three cynomolgus macaques (J16012, J16017, and Ji408005). Between the PCR-positive animals, striking differences were observed in the number of positive tissues. J16012 and Ji408005 were only positive in respiratory tract tissues, Ji408005 tested positive in the pharyngeal mucosa, lung, and two lung lymph nodes, while viral RNA was detected in the carinal part of the trachea, lung, and four lung lymph nodes of animal J16012. In the two other PCR-positive macaques (R14002 and J16017), RNA was detected in the respiratory system and the tracheobronchial lymph nodes, but also in cervical and mesenteric lymph nodes, skin, conjunctiva, liver, spleen, kidney, salivary gland, and heart, all organs and tissues that have been described as target tissues in humans [45]. High viral RNA loads were measured in some lymph nodes of these animals, ranging from 2 × 10^5^ to 1 × 10^6^ GE/g of tissue. Also, in the heart of J16017 and R10002, high viral RNA loads (between 5 × 10^3^ and 1.15 × 10^5^ GE/g of tissue) were detected (Supplementary Table S4).

SARS-CoV-2 replication was evidenced by using sgmRNA PCR analysis on all tissue samples that tested positive in previous viral RNA tests. As sgmRNA is synthesized during virus replication, this assay is recognized as an alternative to virus isolation from tissue samples. In Table 2 and Figure 7, the sgmRNA-positive tissues are shown. Despite the virus being no longer detectable in nasal and tracheal swabs from the 4 animals for more than 26 days (R14002), 30 days (J16017), or even 34 days (J16012 and Ji408005), sgmRNA was detectable in the respiratory tract tissues of all four macaques, convincingly indicating that SARS-CoV-2 continued to replicate in these animals after the alleged resolution of infection that was concluded from the negative nasal and tracheal swab samples.

### 3.8. Histopathology

**Figure 8:**
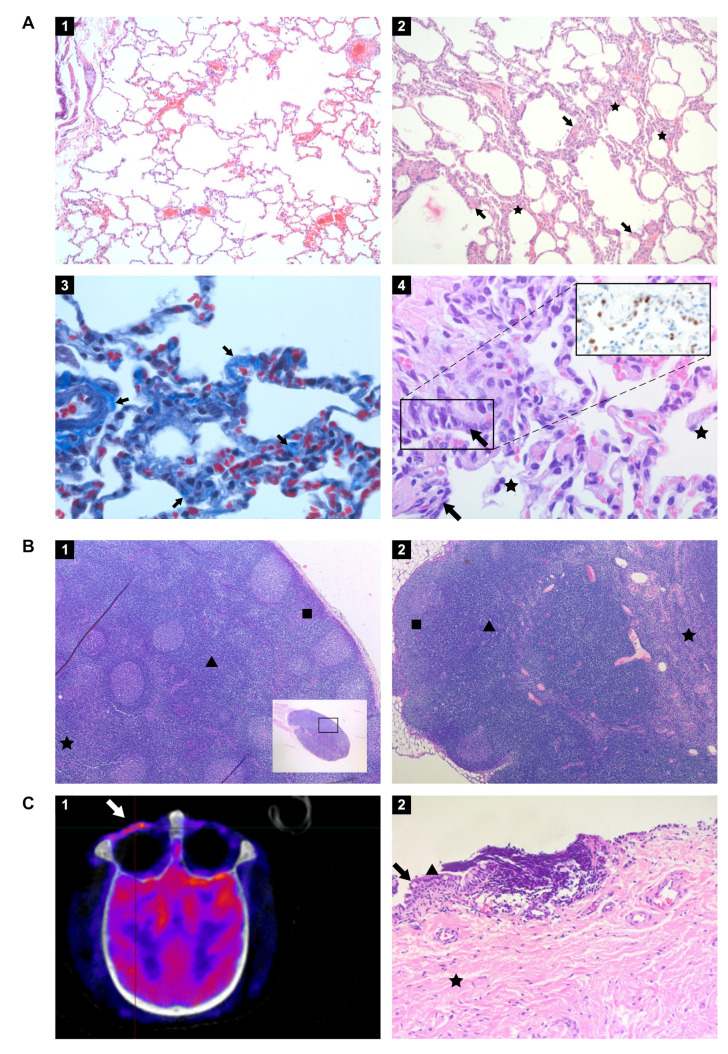
Histopathology of tissues from rhesus and cynomolgus macaques infected with SARS-CoV-2 and euthanized 36 to 42 days post-infection. (**A**) Panel 1. Lung of healthy, uninfected macaque (Hematoxylin and Eosin (HE) staining, original magnification 100×); Panel 2. Lung of rhesus macaque infected with SARS-CoV-2 and euthanized day 36 pi. Focal area with mild alveolar septa expansion with a low number of mononuclear inflammatory cells (asterisks) and foci of bronchiolar smooth muscle hyperplasia (arrows) (HE, 100×); Panel 3. Masson’s trichrome staining shows the presence of collagen (lighter blue) mildly expanding the alveolar septa at some areas indicated by arrows. The internal control is the blood vessel on the left side with lighter blue staining of tunica adventitia (Trichrome stain, 630×); Panel 4. Lung of infected rhesus macaque with foci of mildly thickened alveolar walls lined by cuboidal to columnar epithelial cells with hyperchromatic nuclei representing type II pneumocyte hyperplasia (arrows) and few aggregates of macrophages in the alveolar lumina (asterisks) (HE, 400×). Panel 4 inset: TTF1 marker was used to identify and confirm type II pneumocytes, stained in red-brown (IHC, 400×). (**B**) Panel 1. Cervical lymph node of cynomolgus macaque J16017 with marked follicular lymphoid hyperplasia. Numerous lymphoid follicles in the cortex (square), paracortex (arrowhead), and medulla (asterisk) are indicated (HE, 50×); Panel 2. Mesenteric lymph node of rhesus macaque R14002 with marked paracortical lymphoid hyperplasia and medullar sinusoidal histiocytosis. Cortex (square), paracortex (arrowhead), and medulla (asterisk) are indicated (HE, 50×). (**C**) Panel 1. PET-CT image of the skull of J16017 with prominent metabolic activity in the left ocular region (arrow); Panel 2. Conjunctiva of the same individual. Numerous perivascular and multifocal lymphocytes infiltrating the stratified squamous epithelium and the substantia propria. Goblet cell (arrow), non-keratinized stratified squamous epithelium (arrowhead), and substantia propria (asterisk) are indicated (HE, 200×).

The histological examination of the lung tissue samples revealed unremarkable or resolving minimal lesions that were indicative of a previous infection with SARS-CoV-2. The nature of the lesions was similar in both rhesus and cynomolgus macaques and comprised of some or all of the following: focal areas with minimal to mild interstitial mononuclear inflammatory infiltrates, foci of bronchiolar smooth muscle hyperplasia (Figure 8A panel 2), vascular congestion, multifocal mild expansion of alveolar septa with delicate collagen (Trichrome stain; Figure 8A panel 3), and occasional foci of type II pneumocytes hyperplasia and few intra-alveolar macrophages (Figure 8A panel 4). The latter finding was confirmed by IHC using an anti-TTF-1 antibody (Figure 8A, insert panel 4). Gram staining of the pulmonary lobes of all animals was negative. The extrapulmonary organs were histologically mostly unremarkable, or exhibited mild, nonspecific background lesions, such as chronic-active colitis, and enteritis. However, the conjunctiva of cynomolgus macaque J16017 showed multifocal and perivascular foci of moderate lymphocytic infiltrates in the substantia propria (Figure 8C panel 2). Indeed, this finding was supported by PET-CT images (Figure 8C): an increased FDG-uptake was measured in the left eye compared to the right eye of J16017 which is indicative of ongoing SARS-CoV-2-induced pathogenesis. PCR analysis confirmed the presence of viral RNA in the eye of this animal (Figure 7), clearly demonstrating ocular SARS-CoV-2 infection in J16017. In addition, the cervical lymph node of the same animal exhibited marked lymphoid hyperplasia (follicular type) (Figure 8B panel 1). Similar to the conjunctiva, this histological finding was accompanied by the detection of SARS-CoV-2 RNA in this lymph node. Using IHC, the presence of viral antigen could not be established. Rhesus macaque R14002 showed evidence of SARS-CoV-2 infection in the mesenteric lymph nodes by positive PCR and histologically exhibited marked lymphoid hyperplasia—paracortical type (Figure 8B panel 2).

## 4. Discussion

In humans, COVID-19 displays a broad spectrum of disease symptoms [3,46]. To investigate factors leading to the different COVID-19 manifestations animal models are essential as the moment and site of infection can be controlled and the subsequent response can be monitored in detail. Due to their similarity to humans, NHPs play a pivotal role in this type of preclinical research. To best appreciate the potential of the various macaque species as SARS-CoV-2 infection models, a thorough characterization of the entire course of infection is needed. The comparative study reported here contributes to this knowledge and further validates the different macaque models that are currently in use for COVID-19 research.

The study specifically focused on the post-acute phase of the SARS-CoV-2 infection in rhesus and cynomolgus macaques, defined as the 3–4 week period following the disappearance of detectable virus from the nose and throat swab samples. The majority of published COVID-19 NHP studies concentrate on the first two weeks of infection when evidence was found for acute viral interstitial pneumonia and virus clearance from the nose and throat samples and lung lavages [24,27,47,48,49]. As the PET-CTs performed in this study on days 8 and 10 pi revealed an increase in ^18^F-FDG-uptake in the lungs and various tracheobronchial lymph nodes, it was decided to extend the observation period with weekly PET-CTs for all macaques for several weeks (rhesus macaques 35 or 36 days pi, for cynomolgus macaques 38 or 42 days pi, depending on the PET-CT findings).

During the entire study, including the acute infection phase, only mild clinical symptoms were noticed. This sharply contrasts with findings of others that described SARS-CoV-2-induced clinical disease, with clear signs of fever directly following infection, respiratory symptoms, and changes in hematology and clinical chemistry parameters [24,26]. Unbiased measurement of the body temperature and activity of each animal was performed continuously by using telemetry. This is an important asset as in both macaque species a small, but notable elevation in body temperature was recorded in the first two weeks, the period of active virus replication. Differences in animal activity indicated that SARS-CoV-2 infection also influenced the well-being of the animals without causing obvious clinical symptoms. No indications for renal involvement or coagulation disorders were found in the macaques, and C-reactive protein (CRP) levels, a marker for pneumonia in humans [37], were also not afflicted. The clinical findings deviate from those described by other researchers. This discrepancy may be due, for instance, to the virus strain used for the challenge, the methods used for virus inoculation, the origin, genetic background, and environmental conditions of the animals.

In COVID-19 patients, the acute respiratory syndrome sometimes coincides with hypercytokinemia, occasionally resulting in multi-organ failure [50]. Patients with severe COVID-19 had significantly elevated plasma levels of proinflammatory cytokines [51]. In this study, macaques did not show overt disease symptoms but levels of certain cytokines, such as IL-6, IFN-*γ*, MIP-1α, and MIP-1β increased in the plasma of both macaque species, showing that the chemokine system is involved in SARS-CoV-2 infection. The cytokine profiles after SARS-CoV-2 were highly comparable between the macaque species, except for IP-10 and MCP-1, suggesting differential involvement of monocyte activation between the species. We observed an increase in IL-6 levels at the end of the study in some individuals. In humans, IL-6 is involved in many inflammatory diseases. In patients with COVID-19, systemic production of IL-6 is associated with disease progression [52]. Also, in our study, the elevation of IL-6 corresponded with lung pathology as indicated by the highest CT/pathology scores in these animals. In humans, higher IL-6 levels coincide with increased CRP. We did not observe this correlation in the macaques, underlining subtle differences between human and NHP pathology in response to SARS-CoV-2 infection.

Antibody responses were detectable in all animals after infection. IgG titers to the N and S proteins, as well as the receptor-binding domain (RBD) on S, started to develop around day 14, and apart from individual differences, continued to rise during the follow-up period. The development of IgG accurately followed the course of virus infection, as it became first detectable within one week after the virus had become undetectable in sera by RT-PCR. This was in line with the findings of others [25,32,53]. No IgM directed to the N protein was detected in seven out of eight animals. This result was confirmed by a second, in-house developed IgM-ELISA (not shown) and thus a technical flaw in the serological assay used was excluded. We cannot explain the lack of N-specific IgM response to infection. In contrast, elevated antibody levels against S and the RBD were measured, most prominent in the cynomolgus macaques. Overall, IgM titers developed simultaneously with IgG, a salient phenomenon also reported by Hartman et al. in SARS-CoV-2-infected African Green monkeys [53].

Imaging was used to visualize possible lung pathology. In humans, CT is also applied, but the type of lung lesions found are only partly specific to COVID-19 [3,54]. In the current study, purpose-bred NHPs with a well-documented health status were used, and pre-infection control scans were made. In this well-controlled NHP study, CT imaging is likely a valuable tool to monitor the progression of COVID-19-related lung pathology. Based on the criteria set to determine clinical severity [55], the macaques featured moderate disease levels as all eight individuals showed levels of pneumonia during the infection. As in other NHP studies [32], lesions were observed in the lungs of infected animals. Differences in the location of lesions between the different studies may be related to different circumstances e.g., the method of administration of the virus into the trachea. Similar to Finch et al. [32], we did not collect bronchoalveolar lavage (BAL) samples in order to avoid unwanted interference with CT imaging [34,53,56,57]. Instead, we collected tracheal swabs for viral load analysis. The viral RNA loads, but also the temporal pattern of RNA detection in swabs samples, were similar to those observed in BAL [23,48,58,59].

PET-CT imaging of the respiratory tract started in the late acute phase of infection and was regularly performed until euthanasia. It became evident that after the assumed resolution of infection, as witnessed by negative testing for viral RNA in nasal and tracheal swabs, clear signs of pneumonia were still present in the lungs, with both sustained and newly formed lesions. Besides, an increased ^18^F-FDG uptake was detectable in the lungs and tracheobronchial lymph nodes of all animals. This indicates that despite mild disease symptoms in the acute phase of infection, and resolved viremia, SARS-CoV-2-induced pathogenesis continued. In addition, the detection of subgenomic messenger RNA in the respiratory tract and lung lymph nodes of 50% of animals implies that replication of SARS-CoV-2 continued unnoticed, and one can only hypothesize that this can cause disease symptoms in the macaques at a later stage. This finding in a recognized NHP model for COVID-19 research is of particular interest because of the growing concern for long-COVID in humans [60]. More than a year after the start of the pandemic, it is evident that humans can suffer from COVID-19-related symptoms, weeks to months after seemingly resolving the infection [5,6,7].

COVID-19 was initially regarded as a respiratory disease, but patients that succumb to this disease can display a complex array of pathologies that cover a broad spectrum of symptoms. In the macaques, viral RNA was also detected in other tissues, such as in the conjunctiva, salivary gland, cervical and mesenteric lymph nodes, heart, and kidney. In COVID-19 patients, these organs can also be affected by SARS-CoV-2, causing extra-pulmonary disease symptoms [46,61,62]. Histopathological findings of lymphocytic infiltrates in the lamina propria of the conjunctiva and lymphoid hyperplasia in a cervical lymph node (both in J16017) correlate with the detection of viral RNA and highlight similarities in the disease course between NHP and humans.

Subtle differences were observed between the two macaque species in reaction to the SARS-CoV-2 infection that may affect disease or pathology, but based on the results presented here, both species are equally useful in COVID-19 research.

PET-CT imaging, in combination with viral RNA and subgenomic messenger RNA detection, revealed new insights in the post-acute phase of SARS-CoV-2 infection in macaques. Our data indicate widespread tissue dissemination of SARS-CoV-2 in individual monkeys and provide evidence for continuing virus replication in lungs and surrounding lymph nodes after alleged convalescence of infection. This finding is intriguing as it has been hypothesized that persistent infection contributes to long COVID-19 in humans [60]. One wonders whether the current worldwide COVID-19 vaccination program will not only eliminate the acute disease but will have a positive effect on minimizing the long-COVID-related pathologies as well.

## Figures and Tables

**Table 1:** CT scores of lung lesions in SARS-CoV-2-infected macaques. Scoring was performed independently by two experienced scientists.

Animals									
**Days pi**		**R14002**	**R15080**	**R15090**	**R15096**	**J16004**	**J16012**	**J16017**	**Ji408005**
	0	0	0	0	0	0	0	0	0
	2	0	0.5	1.5	2	2.5	1.5	0	0
	4	0	0	0	3	0	4.5	0	0
	6	0	0	0	2	0	5	0	4
	8	3.5	0	1.5	2.5	0	5	4.5	2
	10	1.75	2	0	2	2	0.5	2.5	3.5
	12	2.5	2.5	0	1	2	1.5	0	0
	14	1	1.5	0.5	2.5	1	0.5	0.5	2
	16	0	0	0	3.5	3	6.5	1.5	3.5
	22	2.5	0	0	3	3	6	0.5	2.5
	29/30	0	2	3	4	3	4.5	0	5
	36/38	nd	nd	nd	nd	5	6.5	1.5	3

**Table 2:** Determination of sgmRNA copies per gram of tissue. PCR analysis was done on all tissues that tested positive for SARS-CoV-2 RNA in the RdRp gene assay (Figure 7). Only sgmRNA PCR-positive tissue samples above the LLOQ of 20 RNA copies/reaction are indicated.

	R14002	J16012	J16017	Ji408005
Lung, upper right lobe	-	-	-	1.6 × 10^3^
Lung, accessory lobe	-	1.52 × 10^4^	-	-
Lung, upper left lobe	2.5 × 10^4^	-	-	-
Bronchus left	-	-	1.27 × 10^5^	-
Bronchus right	-	-	3.3 × 10^4^	-
Trachea	-	-	1.84 × 10^4^	-
Left paratracheal LN	-	-	4.22 × 10^4^	-
	-	-	8.4 × 10^4^	-
Left hilar (bronchial) LN	6.8 × 10^3^	1.52 × 10^4^	9.6 × 10^4^	-
	-	-	7.4 × 10^3^	-
Subcarinal LN	-	1.11 × 10^5^	1.56 × 10^4^	-
Right hilar (bronchial) LN	-	3.99 × 10^5^	2.4 10^4^	1.98 × 10^4^
	-	-	-	6.88 × 10^4^
Right paratracheal LN	-	1.34 × 10^5^	1.26 × 10^4^	-
	-	-	9.2 × 10^3^	-

## Data Availability

All data are available in the main text or the Supplementary Materials. Correspondence and request for materials should be addressed to EJV (verschoor@bprc.nl).

## Supplementary Materials

The following are available online at https://www.mdpi.com/article/10.3390/v13081673/s1, Figure S1: Body temperatures of all animals, Figure S2: Activity of all animals, Figure S3: Cumulative clinical scores, Figure S4: Clinical biochemistry, Figure S5: Development of SARS-CoV-2 IgM response in rhesus and cynomolgus macaques, Figure S6: Cytokine and chemokine levels in SARS-CoV-2-infected macaques, Figure S7: Quantification of ^18^F-FDG uptake by tracheobronchial lymph nodes, Table S1: List of animals used in the study. R: rhesus macaque, C: cynomolgus macaque. Table S2: Clinical symptoms scoring list, Table S3A: Viral loads in swab and blood samples (RNA genome equivalents/mL), Table S3B: Subgenomic messenger RNA in tracheal and nasal swab samples (sgmRNA copies/mL), Table S4: Viral RNA loads in tissues of infected macaques, Table S5: list of consumables.
