# Supplementary material for: The Post-Acute Phase of SARS-CoV-2 Infection in Two Macaque Species Is Associated with Signs of Ongoing Virus Replication and Pathology in Pulmonary and Extrapulmonary Tissues
Source: Viruses. 2021 Aug 23;13(8):1673. doi: 10.3390/v13081673 (PMC8402919; doi:10.3390/v13081673)
Supplement: Supplementary file 1 [file viruses-13-01673-s001.zip › viruses-1290754-supplementary.pdf]

Supporting information

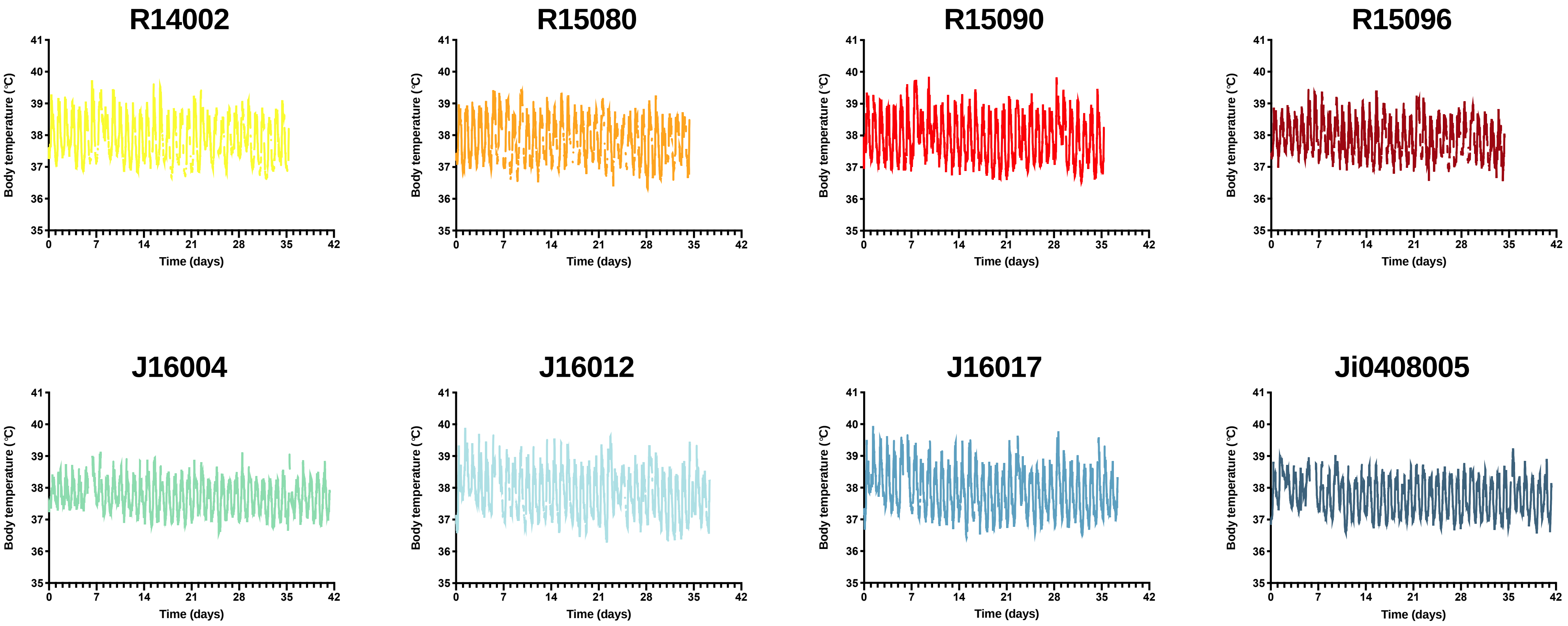

Figure S1. Body temperatures of all animals. Body temperatures were measured using a digital telemetric device during the entire study. The gaps in the graphs are due to data loss during measurement.

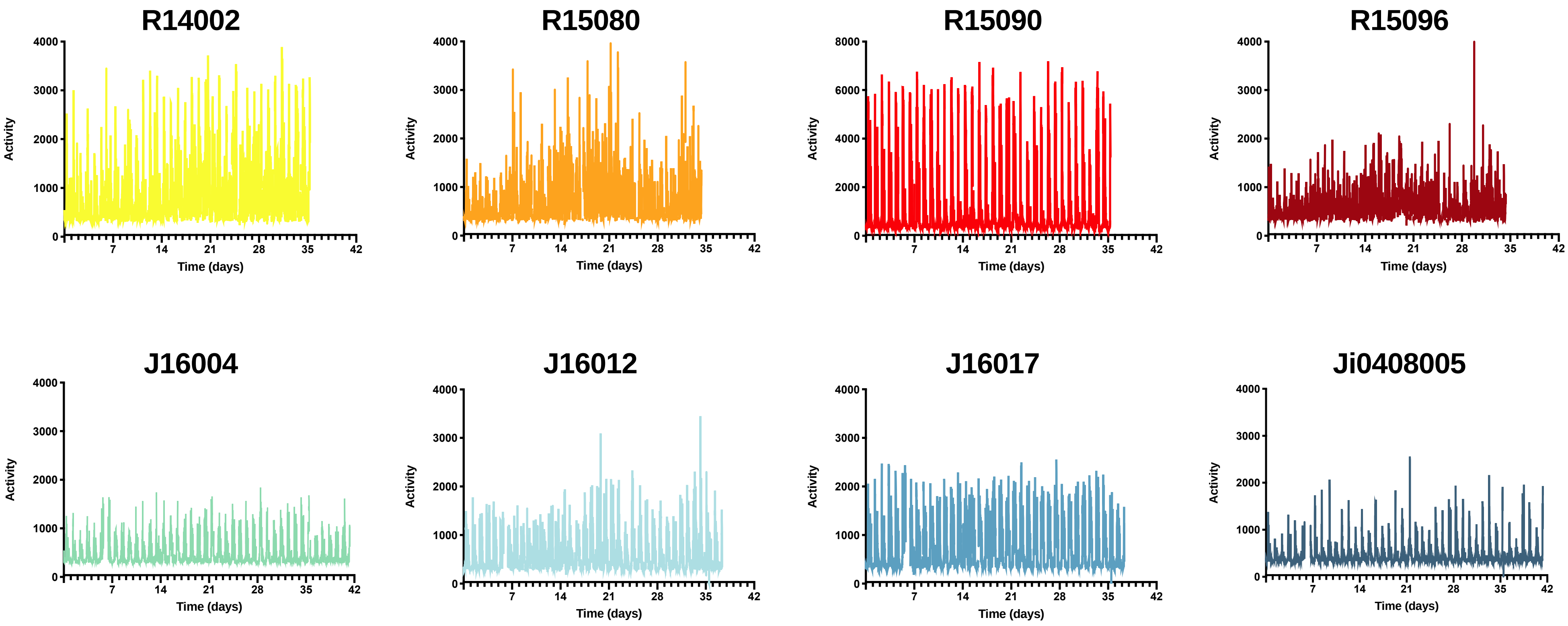

Figure S2. Activity of all animals. (A) The activity of each animal was measured using a digital telemetric device during the entire study. Gaps in the graphs are due to data loss during measurement. **(**B) Cumulative activity scores of the first two weeks compared to the third week of the study, calculated as total area under the curve for the given period.

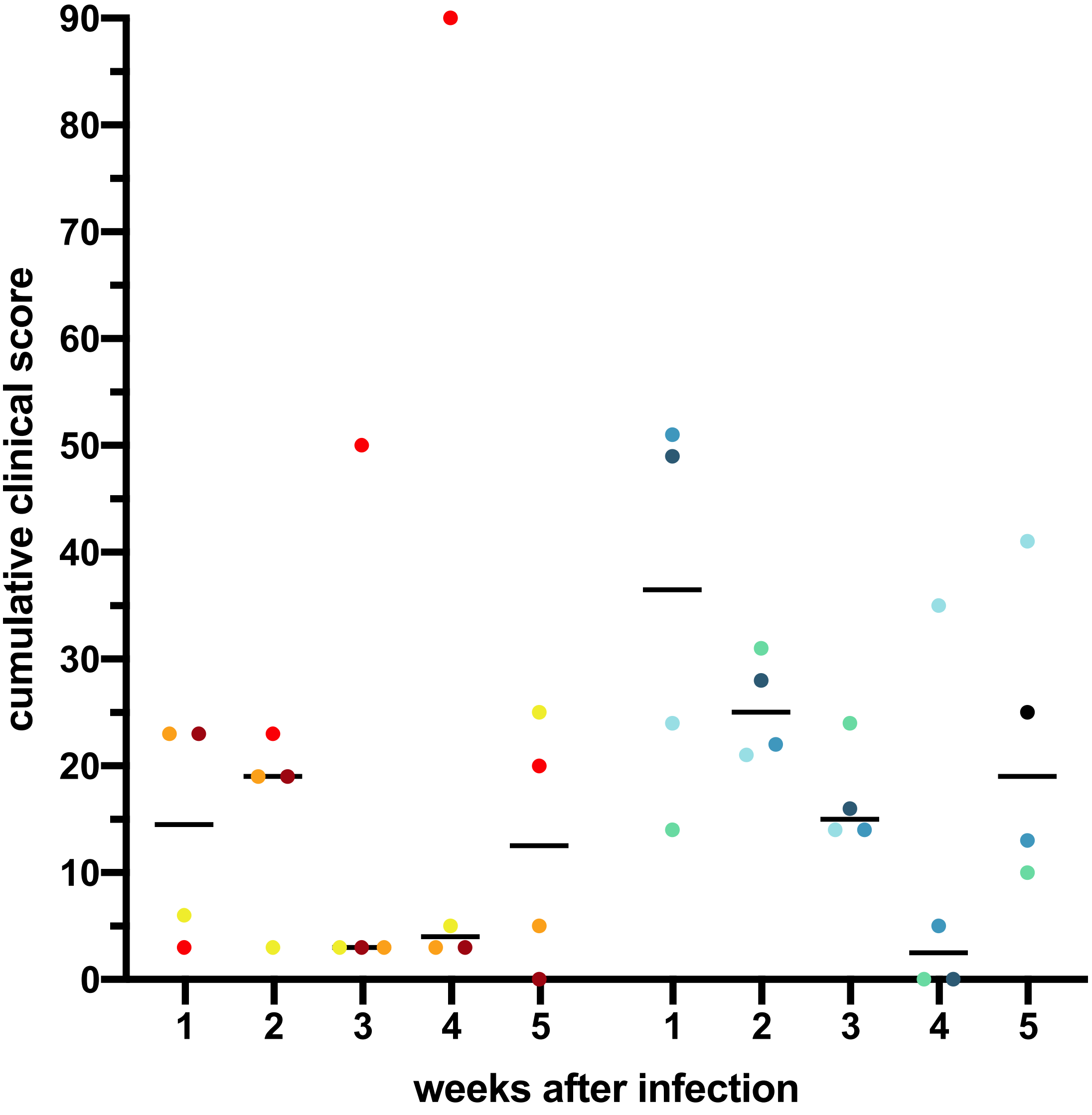

Figure S3. Cumulative clinical scores. Scores were calculated per week and per individual animal. Horizontal bars represent medians.

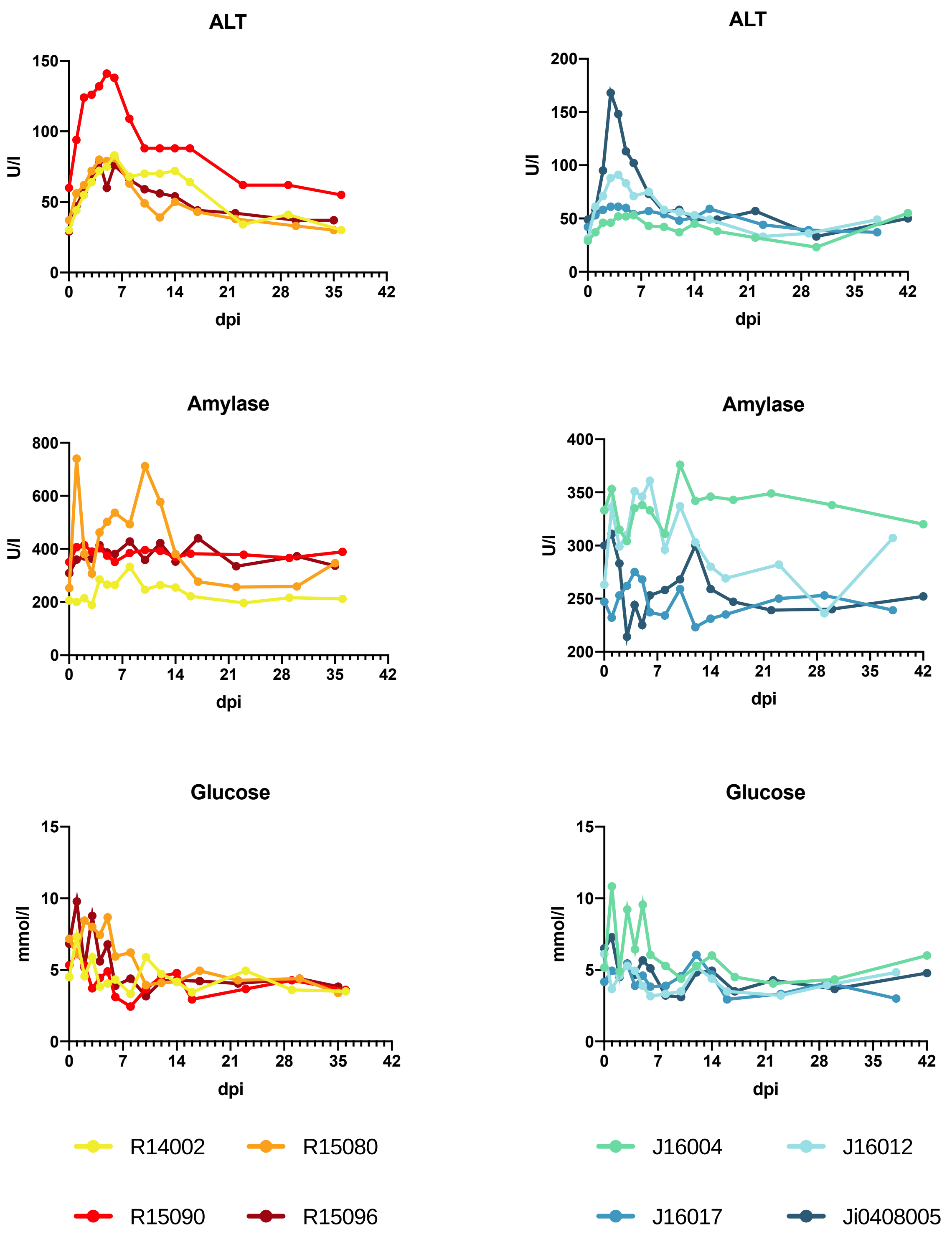

Figure S4. Clinical biochemistry. ALT (alanine aminotransferase), amylase and glucose levels were measured in serum samples of rhesus and cynomolgus macaques

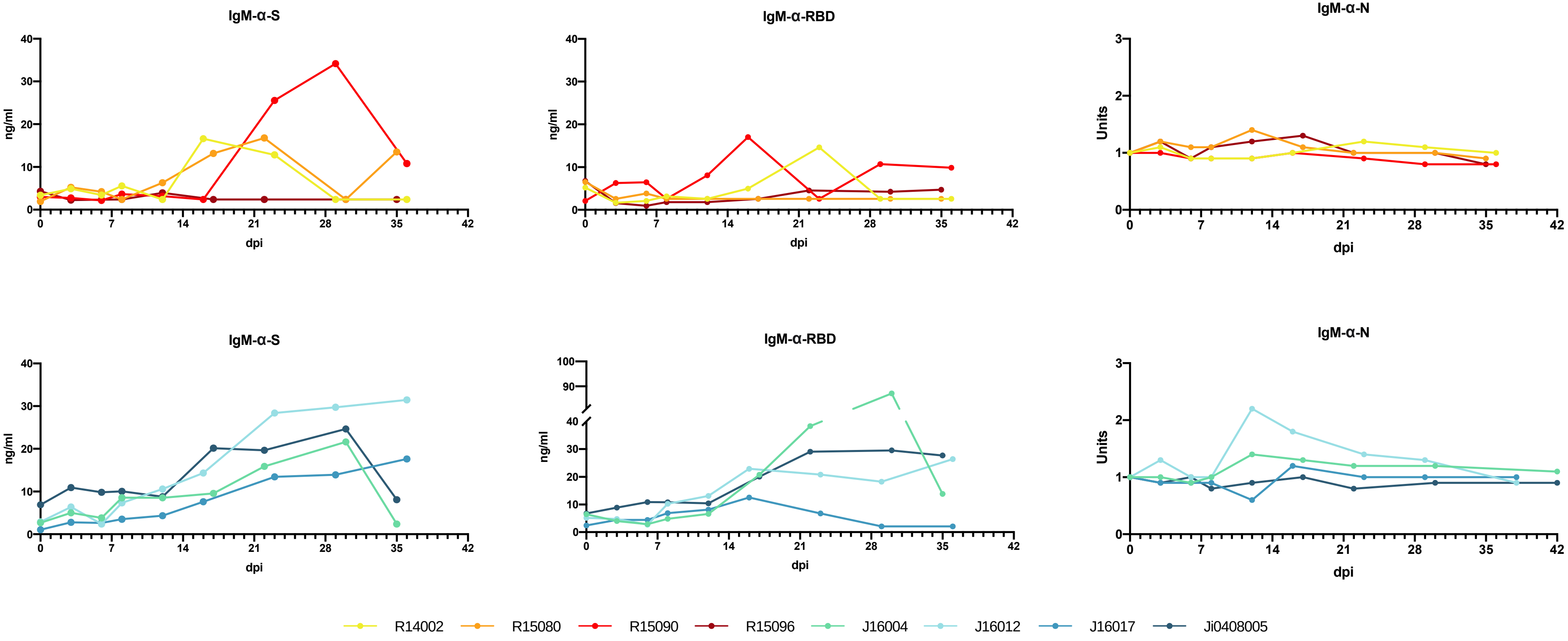

Figure S5. Development of SARS-CoV-2 IgM response in rhesus and cynomolgus macaques. The IgM response in serum was determined using an anti-S IgM ELISA, a serological test to detect IgM directed to the RBD, and an anti-N IgM ELISA (left to right).

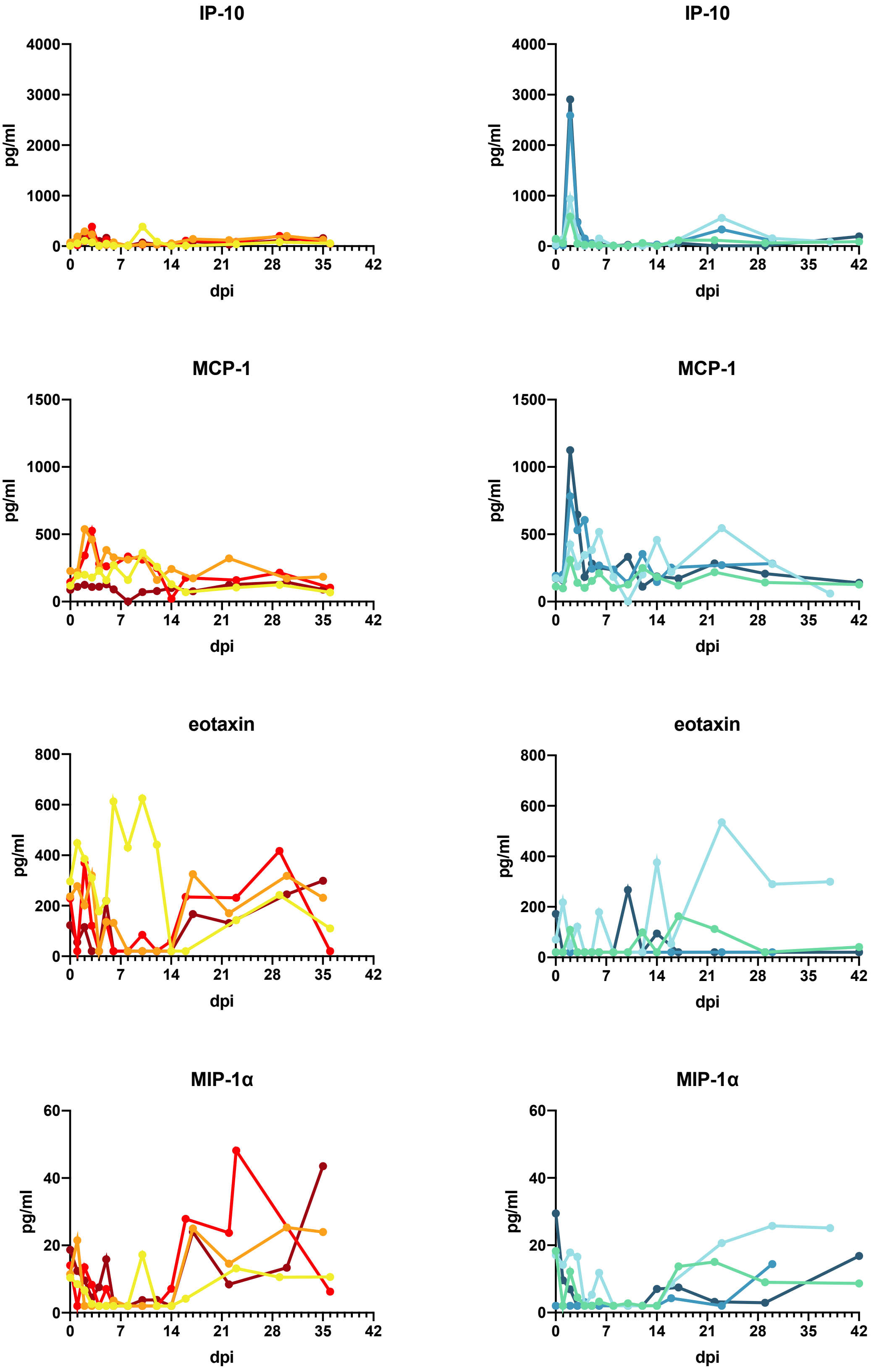
IP-10
IP-10
MCP-1
MCP-1
eotaxin
eotaxin
MIP-1α
MIP-1α
pg/ml
dpi
0
7
14
21
28
35
42
4000
3000
2000
1000
1500
500
800
600
400
200
60
40
20

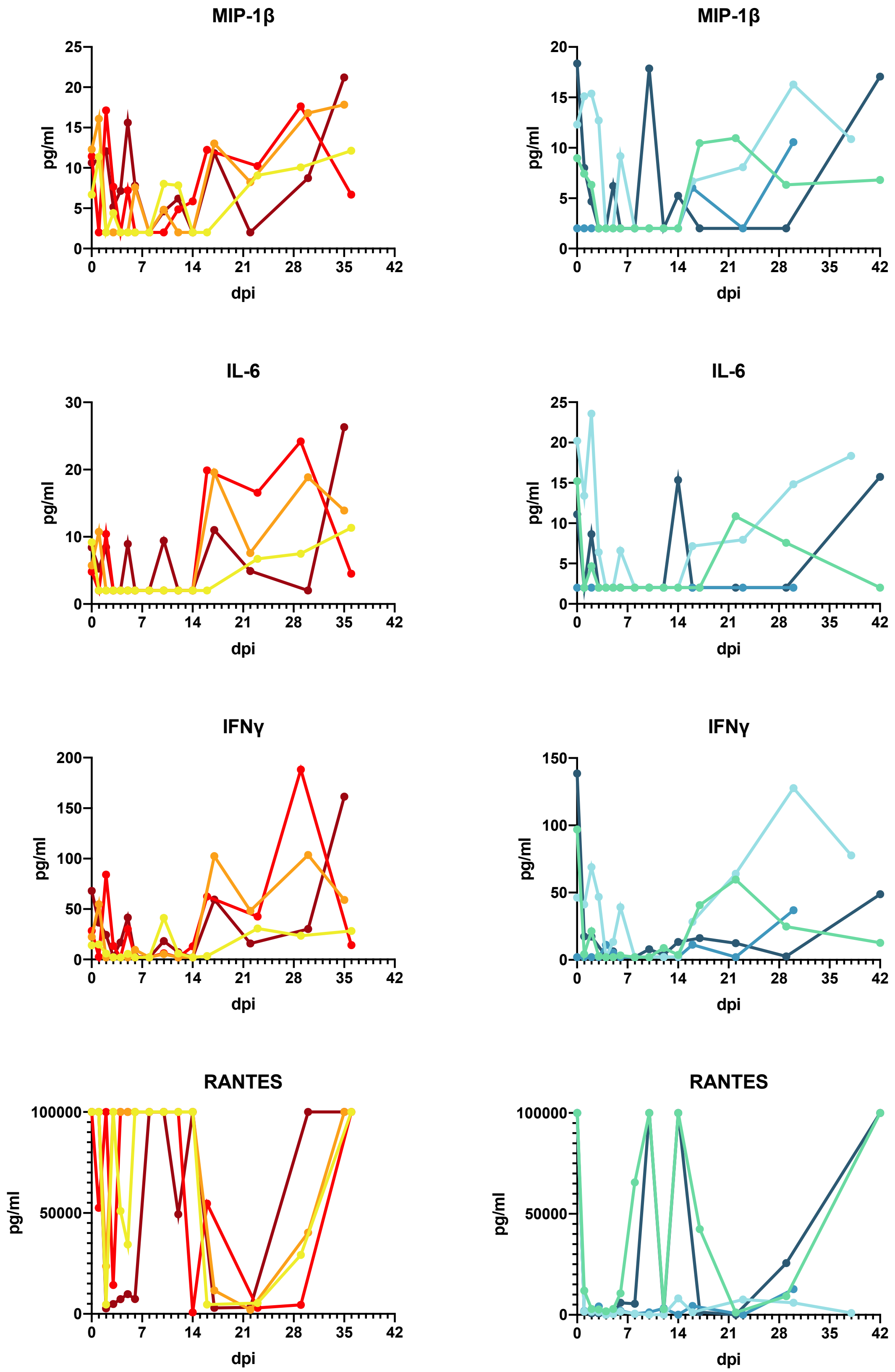
MIP-1β
MIP-1β
IL-6
IL-6
IFNγ
IFNγ
RANTES
RANTES
pg/ml
dpi
0
7
14
21
28
35
42

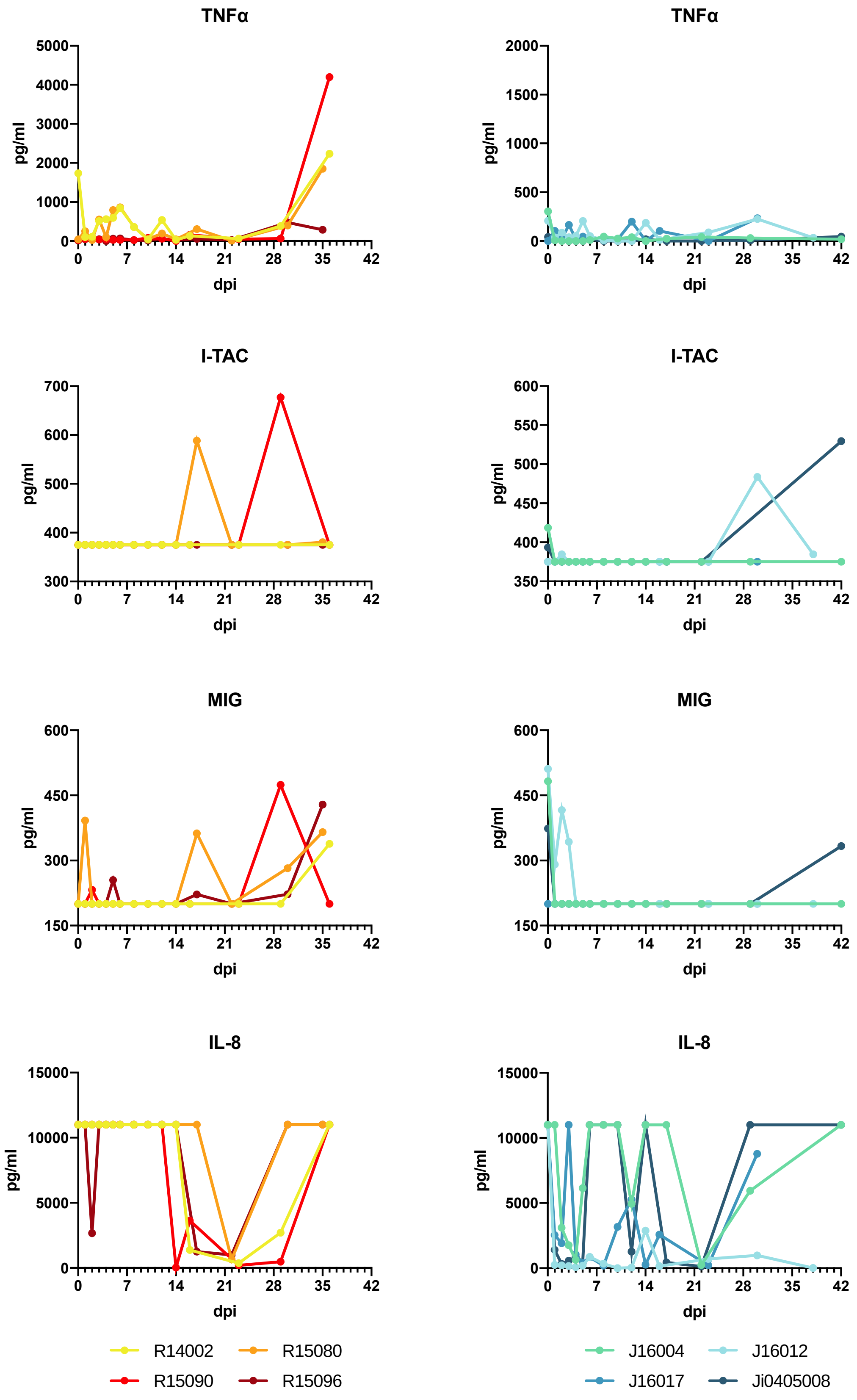

Figure S6. Cytokine and chemokine levels in SARS-CoV-2-infected macaques. Levels were determined using LEGENDplex™ NHP Chemokine/Cytokine Panel (13-plex). Samples were measured on a LSRII FACS machine and analyzed by using company software.

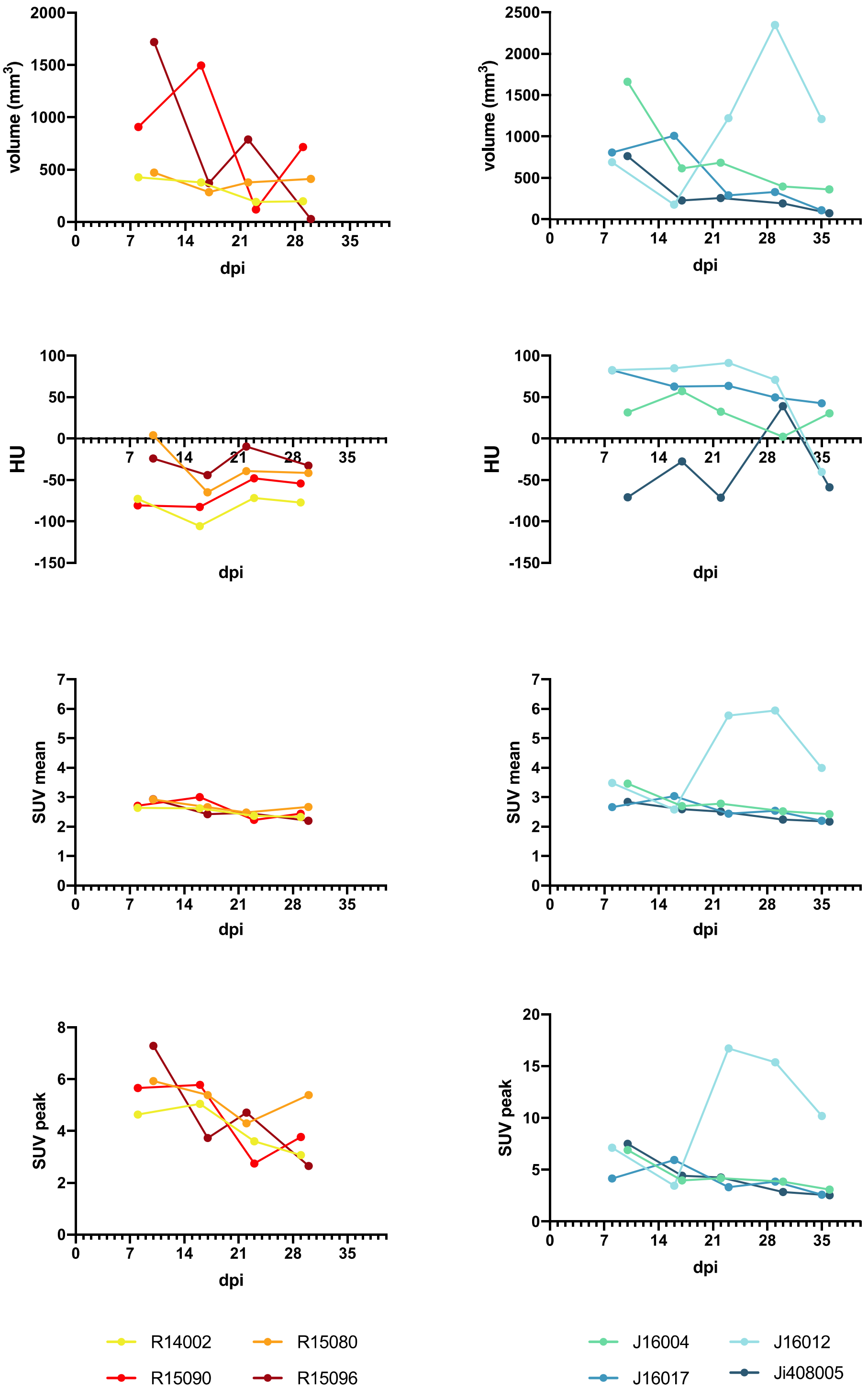

Figure S7. Quantification of ^{18}F-FDG uptake by tracheobronchial lymph nodes. The tracheobronchial lymph nodes were quantified on PET-CT with two anatomical (upper rows) and two metabolic parameters (bottom rows). The anatomical parameters are; volume in mm^3 and density in Hounsfield Units (HUs). The metabolic parameters are the average metabolic uptake represented by the mean standard uptake value (SUV) and the maximum uptake in a region of interest (ROI), corrected for random and scattered coincidences represented by the SUVpeak.

Table S1. Macaques used in study.

Species	monkey ID	Age	Body weight#	Sex
R	R14002	6y	8.2 kg	male
R	R15080	5y	7.9 kg	male
R	R15090	5y	7.8 kg	male
R	R15096	5y	8.7 kg	male
C	J16004	4y	5.7 kg	male
C	J16012	4y	3.3 kg	male
C	J16017	4y	4.9 kg	male
C	Ji0408005	16y	9.7 kg	male

R, rhesus macaque. C, cynomolgus macaque. # weight at start of study

Table S2. Clinical scoring list

Parameter	Observation	Score
**General Appearance**	Normal	0
	Abnormal or Hunched Posture, Dull Appearance to Eyes	5
	Dehydration, Notable Weight Loss, Swelling	10
	Bleeding from any Orifice (not related to routine procedures or menstruation)	15
**Skin and Fur**	Normal	0
	Ruffled Fur, Unkept Appearance,	5
	Rash, Pallor, Redness, Icterus, Petechiae, Ecchymoses, Wound, Abscess, Ulcer	10
**Nose, Mouth, Eyes, Head**	Normal	0
	Nasal Discharge, Excessive Salivation, Ocular Discharge, Lacrimation, Reddened Eyes, Ptosis	5
**Respiration**	Normal	0
	Increased or Decreased Respirations (<30 or >40/min), Cough, Sneezing,	5
	Dyspnea or respiration rate >40/min	15
	Open Mouth Breathing respiration rate >50/min or Cyanosis	35
**Food Intake**	Normal	0
	Decreased (eating less than half the biscuits)	3
	Eating Fruit but no biscuits	5
	Severely decreased (not eating biscuits or fruit)	10
**Locomotor**	Normal	0
	Hyperactivity (circling, increased aggression), Hypoactivity (hunched, inactive through window, active with people in room)	5
	Ataxia, Neurological Signs (tremors, head tilt,), Loss of Interest in Treats	10
	Reluctant to Move, Uses Cage for Support, Difficulty Getting to Food or Water (decreased response to human presence), Seizures	15
	Down with no or Minimal Response to Human Approach, Coma	35

Adapted to Brining *et al*. 2010, Comparative Medicine, Vol 6, No 5, pp 389-395.

Table S3A. Viral RNA loads in swabs and blood (RNA genome equivalents/ml)

**Nose**	day 0	day 1	day 2	day 3	day 4	day 5	day 6	day 8	day 10	day 12	day 14	day 22	euthanasia
R14002	-	4.29×10^{4}	2.57×10^{2}	-	-	-	-	-	-	-	-	-	-
R15080	-	2.32×10^{4}	1.20×10^{3}	4.28×10^{2}	1.80×10^{3}	-	-	-	-	-	-	-	-
R15090	-	9.50×10^{4}	2.20×10^{3}	1.03×10^{3}	2.79×10^{3}	-	-	-	-	-	-	-	-
R15096	-	7.00×10^{3}	-	4.28×10^{2}	-	-	-	-	-	-	-	-	-
J16004	-	3.40×10^{4}	-	-	2.10×10^{3}	-	-	-	-	-	-	-	-
J16012	-	4.30×10^{3}	-	-	2.14×10^{3}	-	-	-	-	-	-	-	-
J16017	-	1.50×10^{4}	-	-	-	9.86×10^{2}	2.10×10^{3}	2.14×10^{3}	-	-	-	-	-
Ji40805	-	1.50×10^{3}	4.90×10^{4}	1.24×10^{3}	-	-	4.71×10^{2}	-	-	-	-	-	-
**Throat**	day 0	day 1	day 2	day 3	day 4	day 5	day 6	day 8	day 10	day 12	day 14	day 22	euthanasia
R14002	-	7.00×10^{4}	1.43×10^{5}	1.17×10^{6}	2.45×10^{5}	1.16×10^{7}	3.19×10^{6}	4.25×10^{6}	3.58×10^{5}	-	-	-	-
R15080	-	2.00×10^{5}	2.40×10^{6}	5.00×10^{4}	4.29×10^{4}	-	-	-	-	-	-	-	-
R15090	-	2.90×10^{5}	1.40×10^{5}	2.40×10^{4}	7.03×10^{3}	3.17×10^{3}	-	-	-	-	-	-	-
R15096	-	1.70×10^{4}	-	-	-	-	-	-	-	-	-	-	-
J16004	-	8.00×10^{4}	4.10×10^{4}	6.40×10^{3}	3.86×10^{2}	-	-	-	-	-	-	-	-
J16012	-	5.00×10^{4}	2.50×10^{5}	-	-	-	-	-	-	-	-	-	-
J16017	-	2.17×10^{6}	1.80×10^{8}	1.50×10^{5}	9.30×10^{6}	2.40×10^{5}	8.83×10^{3}	-	-	-	-	-	-
Ji40805	-	2.70×10^{4}	9.10×10^{4}	5.14×10^{2}	-	-	9.43×10^{2}	-	-	-	-	-	-
**Anal**	day 0	day 1	day 2	day 3	day 4	day 5	day 6	day 8	day 10	day 12	day 14	day 22	euthanasia
R14002	-	-	-	-	-	-	-	-	-	-	-	-	-
R15080	-	-	-	-	3.43×10^{2}	1.29×10^{2}	-	-	-	-	-	-	-
R15090	-	-	-	-	-	-	-	-	-	-	-	-	-
R15096	-	-	-	-	-	-	-	-	-	-	-	-	-
J16004	-	-	-	-	1.76×10^{3}	-	-	-	-	-	-	-	-
J16012	-	-	-	-	-	-	-	-	-	-	-	-	-
J16017	-	3.00×10^{3}	-	-	-	2.10×10^{3}	-	-	-	-	-	-	-
Ji40805	-	-	-	-	-	-	-	-	-	-	-	-	-
**Blood**	day 0	day 1	day 2	day 3	day 4	day 5	day 6	day 8	day 10	day 12	day 14	day 22	euthanasia
R14002	-	-	-	-	-	-	-	-	-	-	-	-	-
R15080	-	-	-	-	-	8.66×10^{3}	-	-	-	-	-	-	-
R15090	-	-	-	-	-	-	-	-	-	-	-	-	-
R15096	-	-	-	-	-	-	-	-	-	-	-	-	-
J16004	-	-	-	-	-	-	-	-	-	-	-	-	-
J16012	-	-	-	-	-	-	-	-	-	-	-	-	-
J16017	-	-	-	-	-	-	-	-	-	-	-	-	-
Ji40805	-	-	-	-	-	-	-	-	-	-	-	-	-

Table S3B. Subgenomic messenger RNA loads in nose and throat swabs (sgmRNA copies/ml)

Nose	day 0	day 1	day 2	day 3	day 4	day 5	day 6	day 8	day 10	day 12	day 14	day 22	euthanasia
R14002	-	-	-	-	-	-	-	-	-	-	-	-	-
R15080	-	-	-	-	-	-	-	-	-	-	-	-	-
R15090	-	-	-	-	-	-	-	-	-	-	-	-	-
R15096	-	-	-	-	-	-	-	-	-	-	-	-	-
J16004	-	-	-	-	-	-	-	-	-	-	-	-	-
J16012	-	-	-	-	-	-	-	-	-	-	-	-	-
J16017	-	-	-	-	-	-	-	-	-	-	-	-	-
Ji40805	-	-	-	-	-	-	-	-	-	-	-	-	-
**Throat**	day 0	day 1	day 2	day 3	day 4	day 5	day 6	day 8	day 10	day 12	day 14	day 22	euthanasia
R14002	-	7.88×10^{3}	3.96×10^{4}	2.07×10^{5}	5.06×10^{4}	1.12×10^{6}	6.74×10^{5}	4.92×10^{5}	4.15×10^{4}	-	-	-	-
R15080	-	3.40×10^{4}	8.65×10^{5}	1.71×10^{2}	-	-	-	-	-	-	-	-	-
R15090	-	3.32×10^{4}	4.64×10^{4}	-	-	-	-	-	-	-	-	-	-
R15096	-	-	-	-	-	-	-	-	-	-	-	-	-
J16004	-	6.17×10^{3}	3.37×10^{4}	-	-	-	-	-	-	-	-	-	-
J16012	-	1.29×10^{2}	2.03×10^{4}	-	-	-	-	-	-	-	-	-	-
J16017	-	3.36×10^{5}	3.94×10^{7}	7.79×10^{4}	3.58×10^{6}	-	-	-	-	-	-	-	-
Ji40805	-	-	1.50×10^{4}	-	-	-	-	-	-	-	-	-	-

Table S4. Viral RNA detection in organs of SARS-CoV-2-infected macaques.

	R14002	R15080	R15090	R15096	J16012	J16017	J408005	J16004
Skin lesion	-	-	-	-	-	1×10^4	-	-
Conjunctiva	-	-	-	-	-	3.88×10^4	-	-
Tongue mucosa	-	-	-	-	-	-	-	-
Nasal mucosa	-	-	-	-	-	-	-	-
Pharyngal mucosa	-	-	-	-	-	7.7×10^4	3.6×10^3	-
Laryngeal mucosa	-	-	-	-	-	1.23×10^5	-	-
Deep cervical lymph node (LN)	-	-	-	-	-	3.4×10^3	-	-
tonsil	-	-	-	-	-	-	-	-
Mesenteric LN	3.08×10^4	-	-	-	-	4.8×10^3	-	-
heart, right atrium	-	-	-	-	-	4.76×10^4	-	-
heart, left atrium	8.82×10^5	-	-	-	-	-	-	-
heart, right ventricle	1.62×10^5	-	-	-	-	1.15×10^5	-	-
heart, left ventricle	-	-	-	-	-	3.52×10^4	-	-
liver	-	-	-	-	-	5.93×10^2	-	-
spleen	-	-	-	-	-	2.13×10^3	-	-
kidney	-	-	-	-	-	1.08×10^4	-	-
jejunum	-	-	-	-	-	-	-	-
ileum	-	-	-	-	-	-	-	-
colon	-	-	-	-	-	-	-	-
Salivary gland	-	-	-	-	-	1.38×10^4	-	-
carina	8.8×10^3	-	-	-	$4.4\ 10^3$	-		-
lung, upper right lobe	-	-	-	-	2.2×10^3	-	5.2×10^3	-
lung, accessory lobe	-	-	-	-	3.08×10^3	-	-	-
lung, middle right lobe	2.74×10^4	-	-	-	-	-	-	-
lung, lower right lobe	4.28×10^4	-	-	-	-	-	-	-
lung, upper left lobe	2.2×10^4	-	-	-	-	-	-	-
lung, middle left lobe	1.9×10^4	-	-	-	-	-	-	-
lung, lower left lobe	6.59×10^4	-	-	-	-	-	-	-
Calf vein	-	-	-	-	-	1.5×10^4	-	-
Bronchus left	-	-	-	-	-	1.5×10^4	-	-
Bronchus right	-	-	-	-	-	3.2×10^3	-	-
trachea	-	-	-	-	-	7.43×10^4	-	-
Left paratracheal LN*	6.43×10^5	-	-	-	-	2×10^5	nd	-
	nd	nd	nd	nd	nd	8.24×10^5	nd	nd
Left hilar (bronchial) LN*	9.82×10^5	-	-	-	7.47×10^3	2.58×10^4	-	-
	4.72×10^3	-	-	-	-	6.02×10^4	-	-
	5.67×10^4	-	nd	-	-	nd	-	nd
	-	nd	nd	-	-	nd	-	nd
	nd	nd	nd	-	nd	nd	-	nd
Subcarinal LN*	-	-	-	-	2.58×10^4	1.14×10^4	-	-
	nd	-	-	nd	nd	nd	nd	nd
	nd	nd	-	nd	nd	nd	nd	nd
	nd	nd	-	nd	nd	nd	nd	nd
Right hilar (bronchial) LN*	9.61×10^4	-	-	-	5.65×10^4	8.52×10^4	4.11×10^4	-
	-	-	nd	-	-	nd	-	-
	-	-	nd	nd	-	nd	-	nd
	nd	nd	nd	nd	nd	nd	8.1×10^2	nd
Right paratracheal LN*	nd	nd	-	nd	5.39×10^4	9.81×10^4	nd	nd
	nd	nd	-	nd	nd	6.47×10^3	nd	nd

Amount of viral RNA is given as genome equivalents per gram of tissue. Only tissue samples that were PCR-positive in at least one of the animals are shown in the table.
*Number of lymph nodes sampled from the respiratory tract varied between individual animals.
nd; not done.

Table S5. List of consumables

name	source	catalog number
ABC-HRP kit	Vectastain	PK-6100
Anti-SARS-CoV-2	Thermo Fisher Scientific	MA1 7403
Anti-Spike-RBD-hIgA (CR3022)	Abcam Ltd	ab278112
Anti-Spike-RBD-hIgG1 (CR3022)	Abcam Ltd	ab273073
Anti-Spike-RBD-hIgM (CR3022)	Abcam Ltd	ab278111
anti-Thyroid Transcription Factor -1	DAKO	M3575
biotinylated rabbit-anti-mouse-IgG F(ab')2	Sigma Aldrich	SAB3701007
BSA	Merck Life Science/Sigma Aldrich	A9647
Copan FLOQSwabs	MLS	M101491
DAB	Vectastain	SK-4150
EDTA	VWR	1084521000
fungizone	Fischer Scientific	15290-018
gentleMACS M tubes	Miltenyi Biotec B.V	130-096-335
Goat-anti-human (H+L) IgG-HRP	Thermo Fisher Scientific	31310
Goat-anti-human- IgM-Peroxidase	Sanbio SBA	2020-05
half-area ELISA plates	Greiner Bio-One	675061
hematoxylin	Brunschwig Chemie BV	H-3404
ketamine hydrochloride	Alfasan	1809284-05
LEGENDplexTM NHP Chemokine/Cytokine Panel	BioLegend	740317
MEM	Thermo Fischer Scientific	31095052
monomeric full-length Spike protein	Expresion Biotech	S2-46-001
monomeric full-length Spike RBD protein	Expresion Biotech	S2-45-001
mouse anti SARS-Cov-2-NP ()	Thermo Fisher;	MA1-7403
normal Goat serum	DAKO	X0907
OptiView DAB IHC Detection Kit	Roche Diagnostics	760700
PBS, pH 7.4	Thermo Fischer Scientific	10010023
penicillin-streptomycin	Life Technologies - Invitrogen	15140122
QIAamp Viral RNA mini kit	QIAGEN Benelux B.V.	52906
SedaStart	AST Farma B.V	19F252
SedaStop	AST Farma B.V	19D122
Stop	DIAsource Immuno Assays SA	SS02-1
TMB	DIAsource Immuno Assays SA	SB04-B
TMB substrate	Life Technologies - Invitrogen	34029
Transcriptor First Strand cDNA Synthesis kit	Roche Diagnostics	4896866001
Tris Base	VWR	1648311
Tween-20	VWR	28.829.296
Tween-20	Merck life Science NV	P1379
xylene	Biosolve Chemicals	24250502
